# The Role of Summer Temperature on Aquatic Insect Diversity at Multi‐Decadal Scales Within the Holocene

**DOI:** 10.1111/gcb.70366

**Published:** 2025-08-05

**Authors:** Ashley M. Abrook, Peter G. Langdon, Gordon N. Inglis, Achim Brauer, Paul Lincoln, Roseanna Mayfield, Antti E. K. Ojala, Celia Martin‐Puertas

**Affiliations:** ^1^ School of Geography and Environmental Science University of Southampton Hampshire UK; ^2^ School of Ocean and Earth Science University of Southampton Hampshire UK; ^3^ GFZ Helmholz Centre for Geosciences Potsdam Germany; ^4^ Department of Geography Royal Holloway University of London Surrey UK; ^5^ School of Bioscience, Faculty of Science University of Nottingham Leicestershire UK; ^6^ Department of Geography and Geology University of Turku Turku Finland; ^7^ Geological Survey of Finland Espoo Finland

**Keywords:** annually laminated lake records, chironomid diversity, climate reconstruction, multi‐decadal resolutions, temperature–diversity relationship

## Abstract

Future anthropogenic warming is suggested to have major impacts on global biodiversity, with freshwater ecosystems particularly under threat. Understanding the role of temperature in impacting freshwater biodiversity is therefore of paramount importance. Previous research suggested that temperature has a limited influence on freshwater diversity across the Holocene. However, this is mostly based on data resolved at centennial to millennial scales, and it is therefore unknown whether freshwater diversity responds similarly over shorter (decadal/multi‐decadal) timescales. We present aquatic insect (Chironomidae) summer temperature reconstructions and key diversity metrics (α‐diversity, β‐diversity, and network skewness) from three annually laminated lacustrine records from Europe (Diss Mere, UK; Nautajärvi, Finland; and Meerfelder Maar, Germany) across the Holocene, including multi‐decadal resolutions within the Holocene Thermal Maximum (HTM) to test this. Our results reveal three major findings with both spatial and temporal elements: (1) At regional (European) scales, using all available data, there is a significant decline in all three diversity metrics with increased summer temperature; (2) at local multi‐decadal scales across the HTM, in the absence of additional major land‐use and eutrophication drivers, summer temperature has only a minor control on aquatic insect diversity (with the exception of a proposed cooling between 6.2 and 5.9 ka BP); and (3) at local low‐frequency timescales, we find a similar relationship, albeit with warm temperatures appearing to promote assemblage stability. As such, temperature and diversity relationships are complex and non‐linear through time and space. We argue that understanding variability in aquatic insect diversity across different temporal and spatial scales is important to consider when assessing the potential for future biodiversity changes under warming climatic regimes in the coming decades.

## Graphical abstract

We show that regionally across Europe during the last 10,000 years, there is a significant negative relationship between summer temperature and aquatic insect diversity. However, at local, low‐frequency intervals of the same period, we observe only a minor relationship between summer temperature and aquatic insect diversity. This same pattern is observed across local, high‐frequency intervals, which contain both warmer average temperatures and greater temperature variability. Together, this highlights complexity in temperature and aquatic insect diversity relationships through time at different scales.

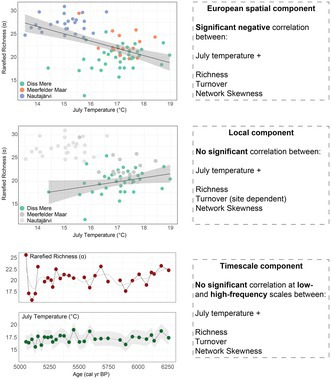

## 1 Introduction

Understanding changing biodiversity patterns at global and local scales is vital for assessments of environmental and societal health (Marselle et al. 2021). Over the 20th Century global biodiversity declined by 2%–11%. With future projected climate and land‐use change, biodiversity losses could increase by 5.1% per decade (Pereira et al. 2024). Freshwater environments are particularly threatened through the direct impacts of temperature and precipitation changes (Rinawati et al. 2013), coupled with associated pressures from exploitation, water pollution, flow modification, destruction, and invasion by exotic species (Dudgeon et al. 2006; Dudgeon 2019; Prakash 2021). The role of temperature in freshwater biodiversity is key, as air temperature influences water temperature, water quality, and chemical constituents (e.g., oxygen solubility), alongside impacting thermal regimes in hydrological systems and niche habitat modification (Hanson et al. 2020; Capon et al. 2021). Any climatically mediated changes to these parameters alter the fundamental biological processes inherent to the populations and species present within the ecosystem (e.g., shifting species range distributions, impacting reproduction, limiting growth, and causing local extinction) (Arneth et al. 2020; Weiskopf et al. 2020; Capon et al. 2021). Given current and projected climate warming during this century, there is a need to better understand freshwater ecosystem responses to past temperature variability in the fossil record where temperatures extend beyond the range of variability in the instrumental period.

Approaches to understand past aquatic insect (Chironomidae) biodiversity patterns have focused on disentangling the influence of past temperature on (1) α‐diversity as a measure of taxon richness (e.g., Engels et al. 2019), (2) β‐diversity as compositional turnover (e.g., Engels et al. 2019; Mayfield et al. 2020, 2021), (3) compositional disorder as a metric for nestedness/unpredictability (e.g., Doncaster et al. 2016), and (4) network skewness (Wang et al. 2019) which measures taxon connectivity (Mayfield et al. 2020, 2021, 2022). The results of these analyses demonstrate a complex chironomid temperature–diversity relationship across different time periods.

In regional modern chironomid calibration datasets from across the northern hemisphere, summer temperature appears to be a strong driver of chironomid α‐diversity at low‐ and mid‐temperature (4°C–14°C) ranges (Engels et al. 2019; Mayfield et al. 2020). This is due to increasing temperature creating suitable thermal windows for a range of different chironomids, but also driving an increase in food availability, complexity of macrophyte communities, and habitat availability (Engels et al. 2019). However, at temperatures above 14°C–15°C this relationship becomes equivocal as richness shows increased variability, plateaus, and/or declines (Engels et al. 2019). This may reflect temperature as a stressor at the warm end of the gradient (e.g., Mayfield et al. 2020, 2021) and/or the influence of associated environmental feedbacks (e.g., oxygen solubility at higher temperatures). Equally, Mayfield et al. (2020) identified complexities in β‐diversity across modern climate gradients, but at mid‐temperature ranges identified general assemblage stability. Mayfield et al. (2022) demonstrated that these same diversity metrics can be particularly sensitive to taxonomic connectivity (i.e., network skewness) and suggested that strongly connected, homogeneous networks (taxa with a large number of interactions) offer enhanced resilience to environmental stressors as minor perturbations have little impact. Equally, where hierarchical structures exist (those where few taxa demonstrate connectivity) perturbations that affect the less connected taxa would be buffered in the system; also demonstrating resilience (Zheng et al. 2024).

In fossil datasets, across critical climatic transitions (e.g., between stadial and interstadial periods), summer temperature drives changes in both α‐diversity and β‐diversity. Over these phases, transitions from warm to cool stadial climates reveal a decrease in taxon richness, as warm taxa are lost from the assemblage, while the amplitude of climate change is intrinsically linked to the amount of compositional turnover (Engels et al. 2019; Mayfield et al. 2022). However, over these transitions, network skewness has been shown to increase (Mayfield et al. 2022). This may result from reduced competition for habitat availability, following both reductions in richness and changes to taxon connectivity (Mayfield et al. 2022).

In contrast, in the Holocene, where climatic transitions are of relatively smaller magnitude, patterns in richness and turnover are difficult to identify (Engels et al. 2019) alongside an absence of any trend in network skewness. This suggests minimal changes to ecosystem diversity or structure (Mayfield et al. 2022). Hence, it is often argued that muted temperature change during the Holocene exerts limited influence over the temperature–diversity relationship (Velle et al. 2005; Engels et al. 2019; Mayfield et al. 2021). In the Holocene, local site‐specific factors (e.g., soil and vegetation development, water quality, nutrient availability, and lake pH) are considered more important than climate directly. However, many Holocene chironomid records are resolved to multi‐centennial scales, with few that offer higher data resolutions (e.g., Velle et al. 2005; Luoto and Sarmaja‐Korjonen 2011; Andrén et al. 2015; Self et al. 2015; Solovieva et al. 2015). The lack of highly resolved records, containing the suite of metrics outlined above, prevents the identification of multi‐decadal patterns in diversity and/or differences in the temperature–diversity relationship that are relevant for policy decision‐making given expected future climate change.

Here we use European annually laminated (varved) sedimentary records, which exhibit excellent chronological precision, to produce a multi‐site comparison of highly resolved (multi‐decadal to sub‐centennial) chironomid‐inferred July temperatures and diversity metrics (α‐diversity, β‐diversity, and network skewness) across the Holocene Thermal Maximum (HTM; ca. 6.5 ka BP). We evaluate whether chironomid‐inferred temperature reconstructions and diversity metrics exhibit similar relationships across different climate regimes in Europe. Additionally, we assess whether these relationships vary with sample resolution by comparing observations from high‐ and low‐frequency intervals (e.g., Engels et al. 2025). Finally, we consider whether individual cooling episodes during the HTM (e.g., at 5.9 and 5.3 ka BP; van Dijk et al. 2024) elicit an ecological response. We focus on the HTM as proxy‐derived reconstructions are warmer than pre‐industrial conditions (Kaufman et al. 2020; Erb et al. 2022). This interval may therefore be relevant for understanding temperature–diversity relationships under future warming scenarios.

## 2 Materials and Methods

### 2.1 Site and Sediment Stratigraphy

**FIGURE 1:**
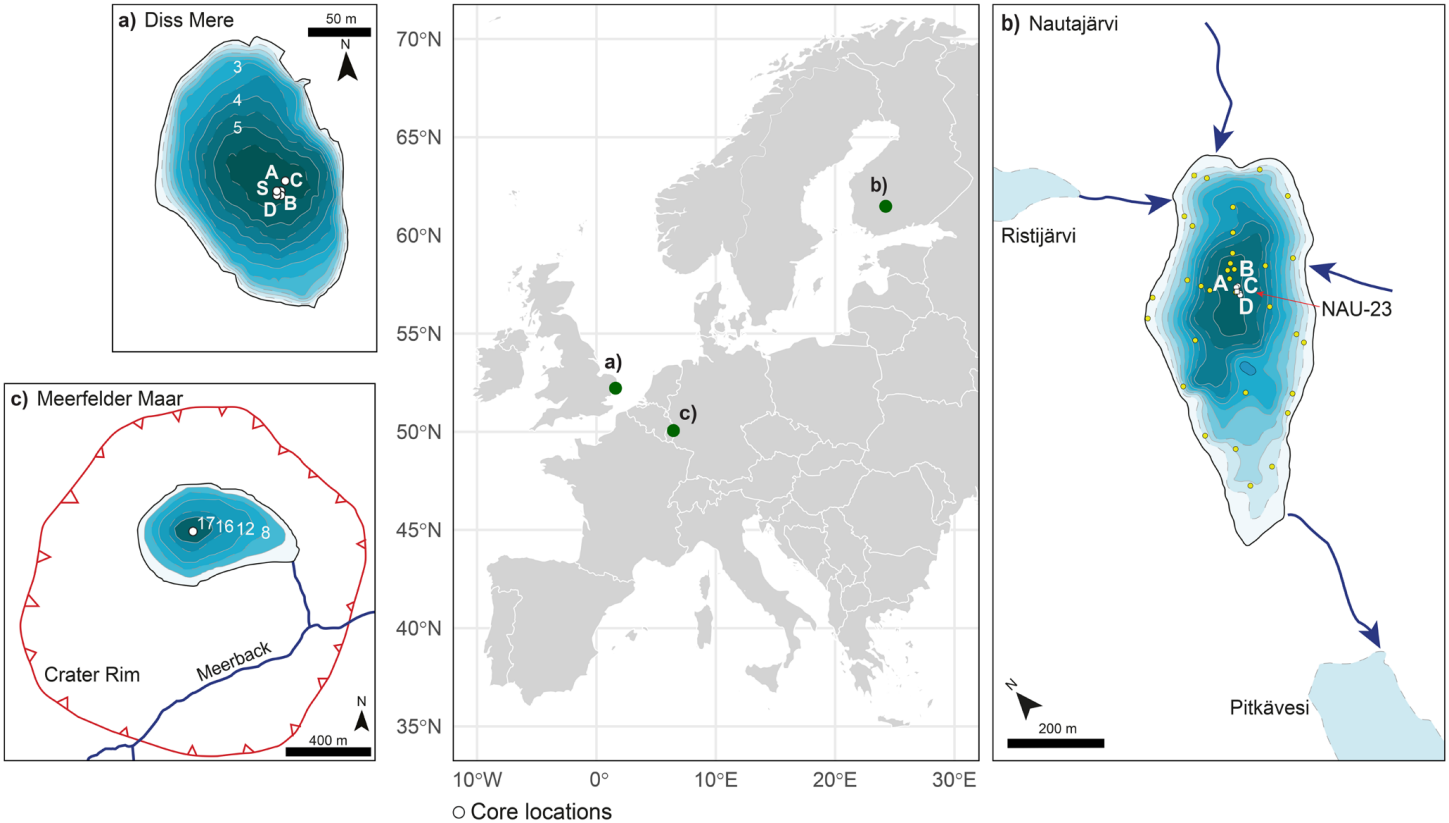
Site locations. (a) Bathymetry of Diss Mere, East Anglia, UK, letters show the location of the DISS‐16 sequence, modified from Boyall et al. (2024); (b) bathymetry of Nautajärvi, Finland with location of the NAU‐23 sequence, alongside surrounding lakes and inflows/outflows, modified from Lincoln et al. (2025); (c) Bathymetry of Meerfelder Maar, Germany and the location of the MFM‐09 sequence, redrawn from Martin‐Puertas et al. (2017).

To investigate temperature and chironomid diversity in NW Europe, we use three annually laminated sediment records from lakes in England, Finland, and Germany (Figure 1). These sites occupy different climatic regions including mid‐latitude maritime climate (England, Western Germany) and high‐latitude cold/cool climate (Finland) which permits the exploration of temperature–diversity relationships across different climate regimes. As these lake sediments are seasonally–annually resolved, they provide the potential for enhanced temporal analyses.

#### 2.1.1 Diss Mere, England, UK

Diss Mere (52°22′ N, 1°6′ E) is a small 6 m deep, presently eutrophic lake with alkaline waters and a chalk bedrock (Martin‐Puertas et al. 2021). The lake contains no inflow or outflow and experiences minimal groundwater input (Boyall et al. 2023). At Diss Mere, we utilize the DISS‐16 sequence, which exhibits 15 m of sedimentation covering the last 10 thousand years, with 8473 varves identified before 2000 years before present (cal. ka BP) (Martin‐Puertas et al. 2021). After 2 cal. ka BP, the sediments are massive and rapidly accumulating, with the lake heavily affected by human activity (Boyall et al. 2024). Varve microfacies analysis shows that the lamina are composed of calcite‐organic couplets, with some interannual variability, and monospecific diatom blooms preceding the calcite layer (Martin‐Puertas et al. 2021). The calcite layer is deposited during summer months, and the organic and diatomaceous layer is deposited during the rest of the year. Diss Mere exhibits thermal stratification in the summer months, leading to hypolimnetic hypoxia at this time (Boyall et al. 2023).

#### 2.1.2 Nautajärvi, Finland

Nautajärvi (61°48′ N, 24°24′ E) is a 20 m deep mesotrophic lake exhibiting slightly acidic lake waters (Korkonen et al. 2017). The site sits in a tiered lake system within the Äväntäjärvi drainage basin and is fed by streams and Ristijärvi to the north and is drained by Pitkävesi to the south (Ojala and Alenius 2005). We use the NAU‐23 stratigraphy (Lincoln et al. 2025) which contains 7.3 m of sedimentation, with 9898 varves (9675 since the isolation from the Baltic Sea basin; Ojala et al. 2005) covering the upper 6.74 m (Lincoln et al. 2025). The varves at Nautajärvi are still forming in the present day (Ojala et al. 2013) and consist of clastic‐organic couplets. The clastic layer forms in spring and is a product of winter precipitation and snow‐melt run off once the lake becomes ice‐free and subaerially exposed. The organic layer forms in the summer months and is principally a function of organic productivity in the water column (Ojala and Alenius 2005; Ojala et al. 2013). Inverse stratification is observed during the winter months at Nautajärvi, leading to persistently anoxic bottom waters (Ojala and Alenius 2005).

#### 2.1.3 Meerfelder Maar, Germany

Meerfelder Maar (50°06′ N, 6°45′ E) is an 18 m deep, eutrophic maar lake that sits within a volcanic crater. The lake has no inflows but contains a small outflow to the Meerback Stream to the southeast of the lake (Brauer et al. 2000; Martin‐Puertas et al. 2012). At Meerfelder Maar, we use the MFM‐09 sequence, which is 11.71 m long, with the upper 7.45 m attributable to the Holocene (Martin‐Puertas et al. 2012). In the Holocene section, varves are preserved, covering the last 11.6 thousand years until ca. 1.5 thousand years ago (Martin‐Puertas et al. 2012, 2017). The varve structures are diatomaceous, with a spring and summer layer composed of a monospecific diatom bloom and the autumn and winter layer composed of both organic and some silt and clay minerogenic detritus (Brauer et al. 2000; Martin‐Puertas et al. 2012). Meerfelder Maar previously exhibited hypolimnetic anoxia, but hypolimnetic treatments have been undertaken to increase benthic oxygen status (Nürnberg 2007).

#### 2.1.4 Chronology

At each site, we utilize the precise, existing chronologies which are derived from annual layer counting and either tied to a radiocarbon or tephra framework (Martin‐Puertas et al. 2012, 2021; Lane et al. 2015; Meerfelder Maar, Diss Mere) or from the cross‐correlation of marker layers between an existing sequence and newly derived sediments (e.g., Ojala and Alenius 2005; Lincoln et al. 2025; Nautajärvi). The stratigraphies and varve‐based chronologies are estimated to have a maximum counting error of ±1% (Ojala and Alenius 2005; Martin‐Puertas et al. 2012, 2021). For Diss Mere and Meerfelder Maar, we present reconstructions as cal. ka BP (hereafter ka BP) and for Nautajärvi, ka varve years BP; all relative to CE 1950.

### 2.2 Chironomid Sampling and Preparation

Across the three composite stratigraphies, samples were extracted for chironomid analysis at variable resolutions. For all chironomid samples, 0.5 cm slices of sediment (2.6–5.2 g) were obtained, resulting in average sample resolutions of 8–15 years per sample. Over horizons where head capsule abundance was low, additional material was obtained from the same sample horizons. Low head capsule abundance was frequently encountered, which is likely a product of hypolimnetic anoxia/hypoxia at each site (see Section 2.1). Principal samples were obtained from the mid‐Holocene (6.5–4.8 ka BP) but to contextualize the Diss Mere and Nautajärvi HTM records, samples were also obtained from sediments attributable to the early Holocene and late Holocene.

Sample processing followed standard procedures outlined in Brooks et al. (2007), with the addition of warm potassium hydroxide (10%) and sieving under 180 μm and 90 μm sieves. All samples were sonicated in a glass beaker for three 10s pulses and re‐sieved to disaggregate and remove unidentifiable organic material. Head capsules were picked and mounted using a Nikon SMZ1000 low power microscope with Hydromatrixan as the mounting medium. Fossil chironomid head capsules were identified using a Brunel Microscopes SP150 high powered binocular microscope at 400× magnification using Wiederholm (1983), Rieradevall and Brooks (2001) and Brooks et al. (2007) as reference material. Given the hypolimnetic hypoxia and anoxic conditions within these lakes, minimum count sums of 40 head capsules were targeted for each horizon. However, this was not always met (Tables S1 and S2). Chironomid diagram construction was performed in R using the “riojaPlot” package (Juggins 2024a) with statistical analysis of the assemblage performed using the “rioja” and “vegan” packages (Juggins 2024b; Oksanen et al. 2024).

### 2.3 Chironomid Temperature Reconstructions

Mean July air temperature estimates were obtained from square‐root transformed fossil chironomid percentage abundance data, from all samples, using the expanded 157‐lake Norwegian calibration dataset (e.g., Brooks et al. 2012) and the combined Norwegian‐Swiss 274‐lake calibration dataset (Heiri et al. 2011). Across both datasets, outlier lakes were removed including 4 from the Norwegian and 19 from the combined training set. For both models, a two‐component weighted‐averaging partial least squares (WA‐PLS; ter Braak and Juggins 1993) regression model was selected. The Norwegian‐Swiss model produced a root mean squared error of prediction (RMSEP) of 1.41°C, a coefficient of determination (*R* ^2^) of 0.86 and a maximum bias of 0.92, estimated by leave‐one‐out cross‐validation. Sample specific errors were calculated using 999 bootstrapping cycles with values ranging from 1.42°C to 1.54°C per sample. All chironomid transfer functions were constructed in R using the packages “analogue” (Simpson et al. 2024) and “rioja” (Juggins 2024b). Model performance statistics are not reported for the Norwegian reconstruction as we use the combined Norwegian‐Swiss as our principal reconstruction in this study (Section 3.1).

### 2.4 Measures of Chironomid Diversity

We follow Engels et al. (2019) in establishing α‐diversity in chironomid samples by calculating taxon richness across the assemblage per individual sample. Here, taxon richness was calculated via rarefaction analysis (e.g., Birks and Line 1992) which considers differences in total head capsule counts per sample. For α‐diversity, all count data were rounded to integers and samples where head capsule counts were below 40 were discarded (as Engels et al. (2019)). To compute α‐diversity, the function *rarefy* was used within the R package “vegan” (Oksanen 2024).

Alongside α‐diversity, we calculate β‐diversity for each sample. β‐diversity, defined as the compositional difference between samples (Legendre and De Cáceres 2013), is a measure of sample dissimilarity. To calculate β‐diversity, we follow the variance partitioning framework outlined in Legendre and De Cáceres (2013) and adopted by Mayfield et al. (2021), where total site β‐diversity (as a function of *Var*(Y) where Y is the community matrix) is established and local contributions to beta diversity (LCBD) are calculated for each sample. High LCBD values reveal dissimilar species compositions between neighboring samples (Legendre and De Cáceres 2013) and is equated with turnover. All samples are used in β‐diversity assessments as tests using only samples with > 40 head capsules produced similar trends. We use the *beta.div* function and Hellinger transformation method prior to analysis within in the “adespatial” R package (Dray et al. 2024).

To investigate ecosystem structures, network skewness was performed on all samples as a measure of connectedness within the chironomid datasets. We compare this measure to the diversity simulations *sensu* Mayfield et al. (2022). Network skewness is defined from network theory as the non‐random associations and interactions between species that co‐occur in the same habitat (Wang et al. 2019). Species interaction is controlled by conditions within the lake/lake functioning/habitat (e.g., nutrient status and lake level) and further external mechanistic controls (e.g., climate and anthropogenic influence). We use the code developed by Wang et al. (2019) in MATLAB (v. 2023a). However, we use the Norwegian‐Swiss calibration dataset to identify co‐occurring taxon pairs with our fossil datasets and use the upper two quartiles (Q2) of positive values for Cramér's association coefficient (V+), following Mayfield et al. (2020), to calculate network skewness. In an unstressed system, positive skewness indicates that a high proportion of taxa have few interactions following taxon self‐organization. A more negative skew value indicates that most taxa have a high number of interactions (with fewer habitat niches) (Wang et al. 2019). Generally, increased taxon connectivity can provide evidence of ecosystem resilience, but greater connectivity can also contribute to critical transitions (e.g., Scheffer et al. 2012; Mayfield et al. 2022).

### 2.5 Data Correlation

To test the relationship between summer temperature, chironomid diversity and structural metrics, correlation matrices, using Pearson's correlation coefficients, were constructed in R using the “ggpubr” and “GGally” extensions to the “ggplot2” package (Kassambara 2023; Schloerke et al. 2024). Correlations were undertaken across a European transect (by combining all data) and for individual datasets at low and high‐frequency intervals. All correlations are performed on samples with minimum counts sums of 40 head capsules (after integer rounding for rarefaction).

## 3 Results

### 3.1 Temperature Reconstructions

Fossil chironomid abundance data are presented in the Appendix S1 and Table 1. Below, we report chironomid temperature reconstructions using the Norwegian‐Swiss model (the Norwegian model is included in the Appendix S1). The combined model performed more strongly in assessments of fit‐to‐temperature, analog qualities (e.g., Francis et al. 2021), and comparability with modern temperatures. This confirms its suitability for temperature reconstructions (Appendix S1) alongside its use in network analysis. Sensitivity tests were also performed with different taxonomic groupings at each site to test the robustness of the temperature signal and to ensure low head capsule sums were not affecting the reconstruction (Appendix S1). While some sample horizons did have head capsule numbers below 40 (Table S1; 1 sample from Diss Mere and 5 samples from both Nautajärvi and Meerfelder Maar had counts < 30), these were included in the reconstruction owing to minimal differences observed during the sensitivity testing approach (e.g., Quinlan and Smol 2001; Bolland et al. 2022).

**TABLE 1:** Main chironomid taxa contributing to each assemblage zone within each site.

Chironomid assemblage zones					
Diss Mere		Nautajärvi		Meerfelder Maar	
Z1	*P. nubesculosum‐*type, *Zavrelymia*, *T. pallidicornis‐*type	Z1	*Sergentia*, *T*. undiff. *Corynoneura*	Z1	*Paratanytarsus*, *P. nubesculosum‐*type, *D. nervosus‐*type
Z2	*C. mancus‐*type, *T*. no spur, *T*. undiff	Z2	*Corynoneura*, *T*. undiff, *Stempelinella*	Z2	*Paratanytarsus*, Instar larvula, *D. nervosus‐*type
Z3	*T*. undiff, *T. pallidicornis‐*type, *Abablesmyia*, *Zavrelymia*	Z3	*Corynoneura*, *T*. undiff, *Heterotrissocladius*	Z3	*P. nubesculosum‐type*, Instar larvula, *D. nervosus* ‐type
Z4	*T*. no spur, *Sergentia*, *Abablesmyia*	Z4	*Stempelinella*, *P. sordidellus‐*type	Z4	*D. nervosus‐*type, *M. pedellus‐*type, *T*. no spur
Z5	*C. intersectus‐*type, *P. nubesculosum‐*type				

*Note:* Zones are not congruent between sites.

**FIGURE 2:**
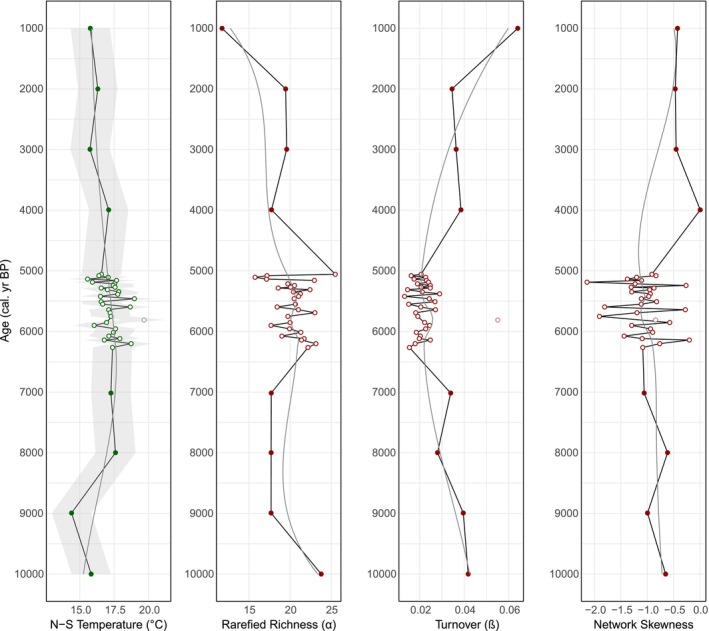
Norwegian‐Swiss summer temperature model and diversity reconstructions (α diversity, β diversity, and network skewness) from Diss Mere across the Holocene. LOESS smoothers have been added to the dataset with a rigid span. The transparent data point at 5.81 cal. ka BP is based on very low head capsule abundance and is unlikely to be robust.

**FIGURE 3:**
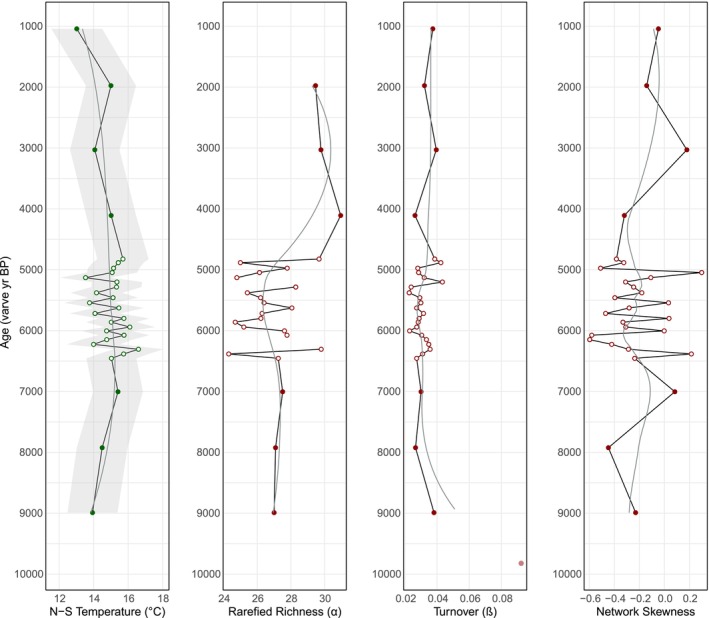
Norwegian‐Swiss summer temperature model and diversity reconstructions (α diversity, β diversity, and network skewness) from Nautajärvi across the Holocene. LOESS smoothers have been added to the dataset with a rigid span. The transparent data point at 9.8 ka varve years BP is based on very low head capsule abundance and is unlikely to be robust.

**FIGURE 4:**
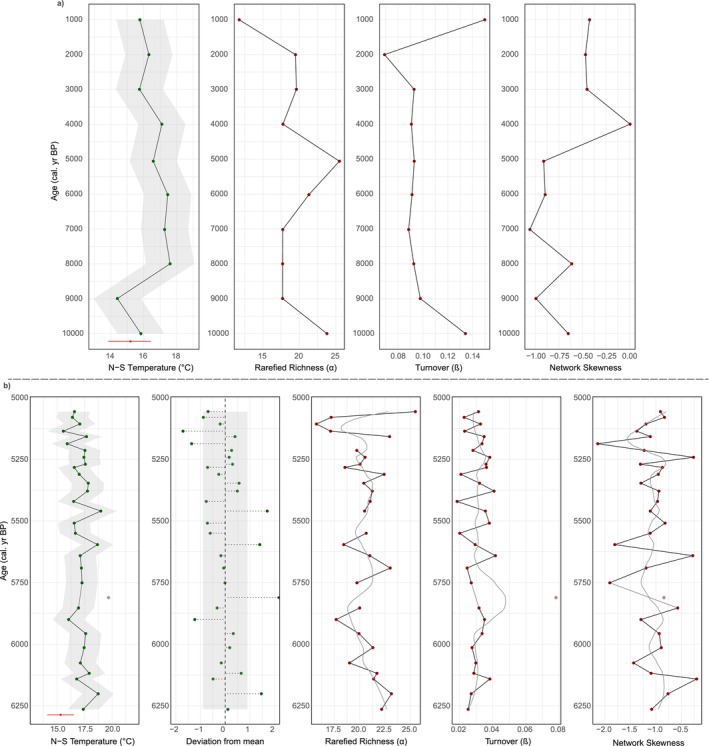
Low‐frequency and high‐frequency summer temperature and diversity reconstructions from Diss Mere. (a) Low‐frequency reconstructions across the Holocene; (b) high‐frequency reconstructions across the mid‐Holocene (6.3–5 ka BP). Gray ribbons on the reconstructed temperatures show the chironomid reconstruction error, while the gray box reveals 1SD from the mean. LOESS smoothers have a flexible span. The red bar displays the median, upper, and lower quartiles of modern temperatures from HadUK gridded temperature data for East Anglia (1961–1990).

**FIGURE 5:**
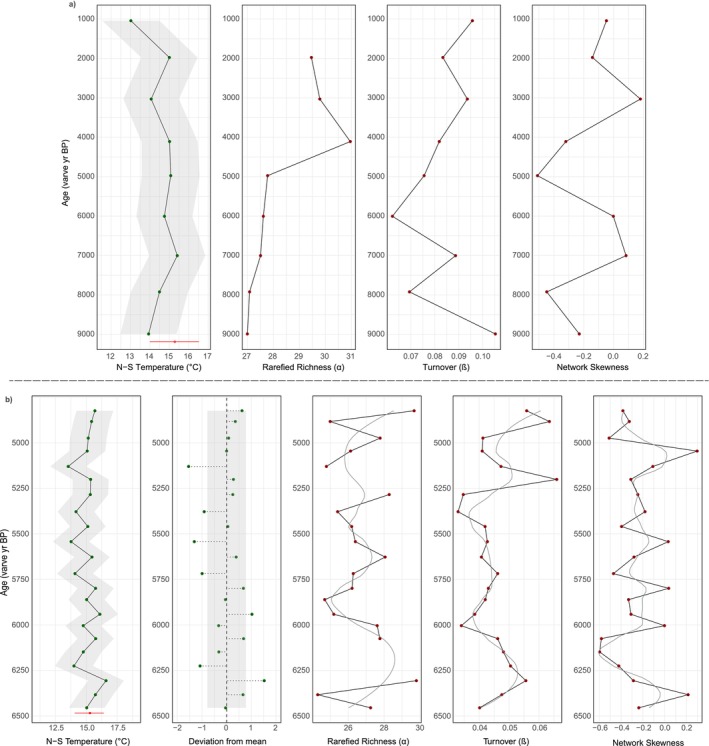
Low‐frequency and high‐frequency summer temperature and diversity reconstructions from Nautajärvi. (a) Low‐frequency reconstructions across the Holocene; (b) high‐frequency reconstructions across the mid‐Holocene (6.5–4.8 ka varve years BP). Gray ribbons on the reconstructed temperatures show the chironomid reconstruction error, while the gray box reveals 1SD from the mean. LOESS smoothers have a flexible span. The red bar displays the median, upper, and lower quartiles of modern temperatures from the Juupajoki Hyytiälä weather station (1961–1990).

**FIGURE 6:**
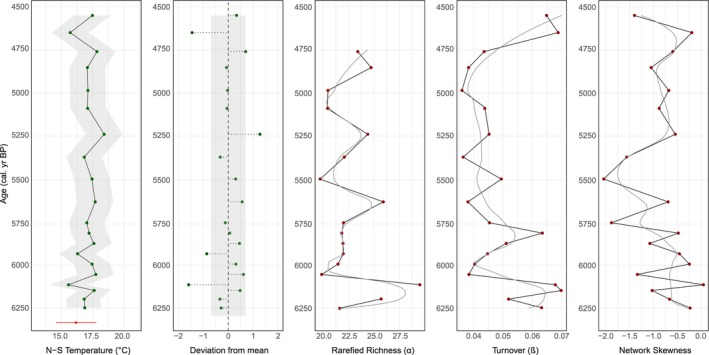
High‐frequency summer temperature and diversity reconstructions from Meerfelder Maar across the mid‐Holocene (6.3–4.5 cal. ka BP). Gray ribbons on the reconstructed temperatures show the chironomid reconstruction error, while the gray box reveals 1SD from the mean. LOESS smoothers have a flexible span. The red bar displays the median, upper, and lower quartiles of modern instrumental temperature from the Manderscheid‐Sonnenhof weather station (1962–1990).

Summer temperature reconstructions are warmest at Diss Mere (14°C–20°C; Figures 2 and 4) coolest at Nautajärvi (13°C–16.6°C; Figures 3 and 5), with Meerfelder Maar being slightly cooler than the former site (15.6°C–18.4°C; Figure 6). Temperature data from Meerfelder Maar are restricted to the mid‐Holocene owing to sediment availability at the time of analysis. Despite differences in absolute values, temperatures gradually increase at Diss Mere and Nautajärvi during the early Holocene, peak during the mid‐Holocene from 6.3 to 5.3 ka BP (Diss Mere) and from 6.3 varve years ka BP (Nautajärvi) and follow a cooling trend into the late Holocene (in agreement with site‐based evidence or regional comparators; Appendix S1). At Diss Mere, the mid‐Holocene record is punctuated by a ~2°C cooling between 6.20 and 5.85 ka BP. The temperature reconstruction from Meerfelder Maar is stable across the mid‐Holocene except for two cooler samples at 6.1 and 5.93 ka BP. Reconstructions at 5.81 ka BP and 9.82 from Diss Mere and Nautajärvi are based on very low head capsule numbers and are not considered robust.

### 3.2 Diversity Metrics‐Richness, Turnover, and Network Skewness

#### 3.2.1 α‐Diversity (Rarefied Richness)

At Diss Mere, richness is variable during the early and mid‐Holocene, with higher richness at 10 ka BP falling and plateauing over the subsequent few thousand years (Figures 2 and 4). Richness oscillates throughout the mid‐Holocene, where high values at ca. 6.20 ka BP fall and then remain variable until peak richness at ca. 5 ka BP. Nautajärvi has the greatest average richness values (Figures 3 and 5) demonstrating greater taxonomic diversity than at the other sites. Peak richness is observed at 4.11 ka varve years BP. At Meerfelder Maar, richness peaks at 6.1 ka BP and oscillates for the remainder of the sequence (Figure 6).

#### 3.2.2 β‐Diversity (Turnover)

At Diss Mere, turnover is highest in the early and late Holocene, with consistently lower values in the mid‐Holocene (Figures 2 and 4). High turnover at 5.81 ka BP is a product of very low head capsule abundance and is not considered a true feature. High turnover at 9.82 ka varve years BP at Nautajärvi is also a product of very low head capsule abundance. The remainder of the Nautajärvi record demonstrates stable turnover (Figures 3 and 5), with the lowest values recorded at 5.38 ka varve years BP. The mid‐Holocene turnover data from Meerfelder Maar (Figure 6) are variable, albeit with the lowest values recorded at 5.36 ka BP.

#### 3.2.3 Network Skewness

All three lakes demonstrate negative skewness. Diss Mere and Meerfelder Maar exhibit the most negative skewness values across the three sequences. At Diss Mere, the data suggest more negatively skewed values in the early and middle Holocene versus less negatively skewed values in the late Holocene (Figures 2 and 4). Meerfelder Maar is negatively skewed throughout, with the most negative values centered around 5.48 ka BP (Figure 6). The Nautajärvi record has the least negatively skewed values of the three locations, with more negatively skewed values appearing in the mid‐Holocene (Figure 5).

#### 3.2.4 Temperature and Diversity Correlations

The temperature inferences from each site are chironomid based, and therefore, the temperature data are not independent of the diversity calculations. However, in the absence of low error climate reconstructions from lake sites, as in Engels et al. (2019), this approach represents one of few methods to investigate the temperature–diversity relationship during the Holocene. All correlations are provided in Table 2. Each of the sites display variable levels of correlation between summer temperature and diversity metrics, which will be explored below.

**TABLE 2:** Pearson's correlation coefficents between reconstructed summer temperature, using the combined Norwegian‐Swiss calibration dataset, and diversity metrics across a European gradient and for individual sites.

	Richness	Turnover	Skewness
European gradient			
All sites, all data	*r* = −0.51; *p* = < 0.001*	*r* = −0.24; *p* = 0.03*	*r* = −0.58; *p* = < 0.001*
Two sites, low frequency	*r* = −0.58; *p* = 0.01*	*r* = 0.08; *p* = 0.74	*r* = −0.45; *p* = 0.06
All sites, high frequency	*r* = −0.54; *p* = < 0.001*	*r* = −0.08; *p* = 0.51	*r* = −0.51; *p* = < 0.001*
Diss Mere			
Whole record	*r* = 0.32; *p* = 0.06	*r* = −0.43; *p* = 0.01*	*r* = −0.23; *p* = 0.17
Low frequency	*r* = 0.12; *p* = 0.74	*r* = −0.35; *p* = 0.32	*r* = 0.08; *p* = 0.82
High frequency	*r* = 0.29; *p* = 0.14	*r* = 0.24; *p* = 0.21	*r* = −0.12; *p* = 0.53
Nautajärvi			
Whole record	*r* = 0.17; *p* = 0.42	*r* = 0.01; *p* = 0.95	*r* = −0.15; *p* = 0.47
Low frequency	*r* = 0.12; *p* = 0.76	*r* = −0.43; *p* = 0.29	*r* = −0.13; *p* = 0.80
High frequency	*r* = 0.39; *p* = 0.1	*r* = 0.30; *p* = 0.21	*r* = −0.16; *p* = 0.52
Meerfelder Maar			
Whole record	*r* = −0.36; *p* = 0.16	*r* = −0.43; *p* = 0.08	*r* = −0.25; *p* = 0.32
High frequency	*r* = −0.36; *p* = 0.16	*r* = −0.45; *p* = 0.07	*r* = −0.25; *p* = 0.32

*Note:* Correlation plots of individual sites can be found in Appendix S1. The asterisk highlights statistically significant correlations.

## 4 Interpretation and Discussion

### 4.1 Regional European and Inter‐Site Diversity Comparisons

**FIGURE 7:**
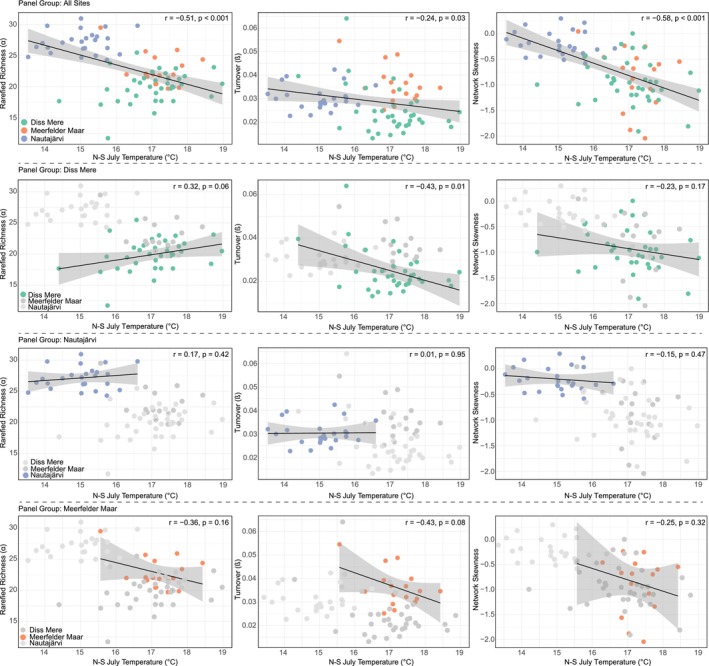
Pearson's correlations of Norwegian‐Swiss July temperatures and richness (α), turnover (β), and network skewness for all data in this study. Green, blue, and orange points relate to Diss Mere, Nautajärvi, and Meerfelder Maar, respectively, with a regression line applied to each correlation. Gray shading reflects the standard error. Low‐ and high‐frequency correlations are provided in the Appendix S1 and in Table 2.

The sites investigated in this study have different local climatic and environmental contexts. However, when viewed from a regional European perspective, i.e., by comparing our data spatially between maritime (Diss Mere, Meerfelder Maar) and high‐latitude continental climate (Nautajärvi) locations, we observe significant negative relationships between summer temperature and each measure of aquatic insect diversity (Figure 7; Table 2). This suggests that increased temperatures reduce α‐diversity, contribute to lower β‐diversity, and produce negative network skew (Figure 7). In this wider assessment, regional climate differences contribute to the observed regional diversity patterns. However, at the site level, factors in addition to climate are important in controlling aquatic insect diversity.

In terms of α‐diversity, Nautajärvi exhibits increased average taxon richness alongside lower reconstructed summer temperatures compared to both Diss Mere and Meerfelder Maar. Previous research has suggested that taxonomic diversity is reduced with cooler climates; for example, with modern reconstructions in N Eurasia (Self et al. 2011) and in the Holocene in Svalbard (Luoto and Ojala 2018). The data from Nautajärvi are therefore counter to this observation. Here, we suggest that increased richness at Nautajärvi was a product of greater niche habitat availability. Support for this can be gained from evidence that Nautajärvi was particularly sensitive in the Holocene with phases of both increased stratification, expanding the hypoxic hypolimnion, and climatically induced strengthening of lake mixing regimes, redistributing nutrients, and dissolved oxygen (see below; Lincoln et al. 2025).

At Nautajärvi, there is greater diversity within the *Tanytarsini* and *Orthocladiinae* sub‐families, which is also observed in alpine lakes at high elevation (Lods‐Crozet et al. 2012). Furthermore, there is a greater abundance of rheophilic taxa (*Rheocricotopus*, *Eukiefferiella*) at Nautajärvi than at Diss Mere and Meerfelder Maar, which provides evidence of an enhanced hydrological influence on the chironomid assemblage (e.g., Nyman and Korhola 2005; Pawłowski et al. 2016). As Nautajärvi sits within the drainage basin of the Äväntäjärvi system and within a series of interconnected lakes, hydrology is an important modulating component of lake conditions. Therefore, with a predominantly sub‐Arctic continental climate, a large seasonal temperature range (Ojala and Alenius 2005) and a large hydrological catchment, it is likely that lacustrine conditions were suitable to support a range of chironomid habitats. In contrast, both Diss Mere and Meerfelder Maar have a much smaller hydrological catchment (Martin‐Puertas et al. 2012; Boyall et al. 2024) which would restrict available habitat types.

Additionally, it is argued that taxonomic richness plateaus or declines with increasing warmth (> 14°C–15°C; Engels et al. 2019; Jackson et al. 2024). Warmer temperatures observed at Diss Mere (average of 17.2°C) and Meerfelder Maar (average of 17.1°C) versus Nautajärvi (average of 14.9°C) perhaps demonstrate the passing of thermal and environmental thresholds, which restrict diversity at the lower latitude sites (e.g., Jackson et al. 2024). This is evidenced by the similarity of the chironomid assemblages, range of temperatures, and richness values (Figure 7; Figures S1 and S3) between Diss Mere and Meerfelder Maar. Warmer average summer temperatures at these sites, alongside increased habitat availability at Nautajärvi, might explain the significant negative relationship between temperature and richness when viewed from a European spatial perspective.

The trends in β‐diversity are comparable across each location, with all sites generally displaying low turnover across the Holocene. It was expected that Nautajärvi would demonstrate considerable variability in compositional turnover, as it exhibits the greatest taxon richness, contains the lowest average head capsule counts, and sits in a higher latitudinal location with a greater annual temperature range (e.g., Smol et al. 2005). But this is not observed here (Figure 7). Despite general similarities in turnover, Diss Mere exhibits greater stability than Meerfelder Maar at similar temperature ranges (Figure 7). We suggest that, as Meerfelder Maar is dominated by *D. nervosus‐*type, *P. nubesculosum‐*type, and *Paratanytarsus* (filter‐feeders, collector gatherers, and scrapers; Grzybkowska et al. 2020; Antczak‐Orlewska et al. 2021) which are associated with macrophytes (Langdon et al. 2010), changes to the abundance of aquatic detritus and aquatic plants influence the chironomid assemblage. This is supported by greater detrital input and enhanced water circulation between ca. 6 and 5 ka BP, indicating enhanced nutrient redistribution and shoreline erosion (Martin‐Puertas et al. 2012). This coincides with changes in the aquatic plant community, with substantial reductions in *Myriophyllum* from 6.3 and 5.8 cal. ka BP, with later increases in *Potamogeton* (Litt and Stebich 1999). We argue that periodic increases in turnover (at 6.1 and 5.8 cal. ka BP) reflect these changes. Here, the plant assemblage appears to directly influence chironomid resilience, with greater turnover and lower resilience under phases of changing macrophyte abundance (Dakos et al. 2015). We suggest that Meerfelder Maar is therefore particularly sensitive (as Brauer et al. 2008) to variability in lake conditions and catchment stability.

All three sites display negative network skewness, suggesting stress within the system and strong taxon connectivity (Wang et al. 2019; Mayfield et al. 2020). Environmental stress on lacustrine systems can cause homogenization and a loss of habitats affecting the network of strongly connected taxa (Wang et al. 2019). Changes to assemblages or individual taxa would therefore cascade to other taxa within the system (Mayfield et al. 2022). We see periods of low turnover as a product of this homogenization. Mayfield et al. (2020) found that temperate lakes produced less negative skewness and that chironomid communities in high‐latitude/Arctic lakes exhibited negative skewness as a product of more extreme environments. We show Nautajärvi as displaying the least negative network skewness, alongside the coolest reconstructed temperatures, which is similar to the cold climate observations from Round Tangle Lake in Alaska (Mayfield et al. 2022). In contrast, Diss Mere and Meerfelder Maar occupy a similar temperature and network skewness envelope (Figure 7).

The lakes studied in this paper are varved systems which experience seasonal hypolimnetic hypoxia/anoxia (Zolitschka et al. 2015) which likely acts as a stressor for benthic/sediment dwelling chironomids (Walker 1987). Therefore, the habitat niche for larval chironomids at the three reported lakes is likely environmentally narrow; within littoral and/or pelagic zones; the latter with chironomids that can survive low oxygen conditions. This relationship would likely be maintained for any lake experiencing seasonal hypoxia. However, less negative skewness at Nautajärvi could indicate that there is relatively less ecological stress on the biotic system (e.g., via greater oxygen availability, consistent hydrological input, lower temperatures/unpassed thermal thresholds and undisturbed varve accumulation) or, given the different hydrology and greater range of mean annual temperatures, enhanced ecological niches (Wang et al. 2019). It is possible, as above, that lower temperatures contribute to a more favorable environment for chironomids; working to reduce stress with fewer strong taxon connections. Indeed, this is suggested by the significant negative correlation between summer temperature and network skewness from the spatial analyses. However, greater habitat availability would also produce less negative skewness as taxa would interact less with the whole assemblage when in their preferred habitats (Mayfield et al. 2022). Lincoln et al. (2025) have argued that over the Holocene, Nautajärvi experienced changes in mixing intensification. Based on trends in Fe, S, and Mn precipitates, Lincoln et al. (2025) suggested that periods of strengthened and weakened overturn cycles during the mid‐Holocene redistributed oxygen into the hypolimnion. This would further reduce ecological stress and create diverse microhabitats, supporting both greater taxonomic richness and less negative skewness. It is likely that a combination of the above features contributed to the patterns observed at Nautajärvi. Given these observations, we suggest increased ecosystem resilience at Nautajärvi (e.g., Dakos et al. 2012; Scheffer et al. 2015) as taxa respond individually in a weakly connected network with buffering provided by the changing regime of the lake.

### 4.2 Temperature and Diversity Relationships Across Different Timescales

#### 4.2.1 Response to Low‐Frequency Temperature Variability Through the Holocene

Over the Last Glacial–Interglacial Transition (LGIT; ca 14.6–10 ka BP) in north‐west Europe, Engels et al. (2019) demonstrated that the principal driver of chironomid diversity was summer temperature. During colder periods of the LGIT, richness decreases, which is driven by taxon loss (e.g., Brodersen and Anderson 2002). During warmer periods, taxonomic richness increases, which is driven by taxon gain (Levesque et al. 1995; Mayfield et al. 2021; Tóth et al. 2022). Likewise, turnover increases immediately following any large climatic shift (Stivrins et al. 2016). These changes relate to the direct and indirect effect of temperature on chironomid physiological processes (Eggermont and Heiri 2012) but also the effect of temperature on modulating lacustrine conditions including nutrient and chemical status (Langdon et al. 2006) and macrophyte and microhabitat conditions (e.g., Płóciennik et al. 2022).

During interglacials the range of temperature variability is reduced (i.e., a lack of temperature declines of > 4°C), therefore the temperature–diversity relationship is less pronounced (Engels et al. 2019; Mayfield et al. 2021). However, the Holocene experienced warm temperature anomalies (e.g., Kaufman et al. 2020) which might be relevant for future diversity responses to climate change. Here, we assess the temperature–diversity relationship across the Holocene by analysis of samples at one‐thousand‐year intervals from Diss Mere and Nautajärvi. Despite the low temporal resolution, the chironomid‐based summer temperature reconstructions broadly agree with local and regional reconstructions (Appendix S1) in Holocene climate evolution.

These data suggest that there is a weak relationship between summer temperature, richness, and network skewness and negative relationships between summer temperature and turnover from both sites (Table 2). The lack of correlation between taxon richness, skewness, and summer temperature over local low‐frequency intervals of the Holocene is not surprising because the amplitude of low‐frequency temperature changes during the Holocene is much smaller than those associated with stadial and interstadial variability (*sensu* Bond et al. 1997; Rohling et al. 2003). Although de Mendoza et al. (2024) also found minor relationships between taxon richness and temperature across transitions in Poland. The lack of a temperature–skewness relationship could indicate relative system stability as taxa fill ecological niches when required (e.g., Scheffer et al. 2012).

Equally, the negative correlation between summer temperature and turnover at both sites reveals that the relative stability of Holocene climate at low‐frequency scales aligns with stable chironomid assemblages. For example, the warmest reconstructed temperatures of the mid‐Holocene, at Diss Mere and Nautajärvi, coincide with consistently low turnover rates. It is likely that low turnover is a product of greater suitability of habitats alongside temperature influences. At Diss Mere, commonly occurring taxa across the low‐frequency range have collector‐gatherer functional traits, which supports ecosystem stability through a consistent organic matter supply (Cao et al. 2024) as observed alongside organic laminations throughout the Holocene. While at Nautajärvi, commonly occurring taxa are those that prefer flowing waters (*Eukiefferiella*, *Heterotrissocladius*), profundal (*Sergentia*, *Psectrocladius*), and littoral areas (*Corynoneura*, *Tanytarsini*) suggesting habitat diversity, which also links to less negative skewness values (Li et al. 2022). It is therefore likely that climate stability at low‐frequency intervals promotes stable assemblages.

A feature of both records is higher turnover and increased network skewness in the late‐Holocene. This occurs with a trend toward lower temperatures and, at Diss Mere, in conjunction with the cessation of varve formation and a shift to eutrophic lake waters (Martin‐Puertas et al. 2021; Boyall et al. 2024). At Nautajärvi, this occurs slightly before pollen assemblage changes post 2.0 ka varve years BP (Ojala and Alenius 2005). These changes are unlikely to be driven by climate, as lower temperatures are likely to reduce stress on the chironomid assemblage. It is more likely that the changes observed here reflect human‐induced changes to the lacustrine system through increasing detrital or organic matter accumulation (e.g., Ojala and Alenius 2005) and influencing the lake trophic state (e.g., Boyall et al. 2024). At Diss Mere, we observe a greater abundance of the eutrophic‐tolerant *Polypedilum* from 2 ka BP, aligning with changes in diversity metrics. At both sites, increased network skewness likely indicates a greater number of weakly connected taxa (Wang et al. 2019) which indicates a complex, self‐organized ecosystem (Albert and Barabási 2002).

At local low‐frequency scales, summer temperature therefore appears to have a relatively minor influence on chironomid diversity.

#### 4.2.2 Response to High‐Frequency (Multi‐Decadal to Sub‐Centennial) Temperature Variability During the Holocene Thermal Maximum (HTM)

Here, we focus on high‐frequency reconstructions within the HTM as (1) increased temperature variability at multi‐decadal and sub‐centennial scales may indicate a different temperature–diversity relationship when compared to lower‐frequency reconstructions (e.g., Engels et al. 2025; and Section 4.2.1); and (2) this period is 0.3°C–1.3°C warmer than pre‐industrial climates (Kaufman et al. 2020) and therefore may act as an analog for future ecosystem responses to climate change. The HTM varied spatially and temporally, though most terrestrial records demonstrate median ages of climatic warmth between 8 and 4 ka BP (Cartapanis et al. 2022). Our reconstructions across the HTM center between 6.3 and 4.8 ka BP.

The data presented within this study during the HTM suggest that there are only weak‐moderate correlations between summer temperature and richness and a reversal to positive associations between summer temperature and turnover at local scales (Table 2). Suggesting that warm climates contribute to mostly stable taxonomic diversity (as Section 4.2.3). As chironomids can respond rapidly to climatic and environmental changes, consistently warm (albeit more variable) temperatures may generate suitable ecological conditions to support a more diverse group of chironomid taxa (e.g., Eggermont and Heiri 2012). However, as the correlations are weak, there are likely other lake‐internal or catchment factors modulating chironomid richness at this time including lake trophic state, redox conditions, mixing regime, organic constituents, pH, and catchment vegetation (Nyman et al. 2005; Luoto et al. 2016). These components though can also be affected by temperature (and precipitation) variability. The lack of correlation between turnover and summer temperature at higher resolutions suggests that high‐frequency temperature changes have a limited role in affecting turnover across the HTM and during warm climate intervals.

The relationship between summer temperature and chironomid diversity is not linear where summer temperatures exceed 14°C–15°C (Engels et al. 2019) as unimodal taxon responses suggest the crossing of climatic/ecological thresholds (Nyman et al. 2005). The average reconstructed summer temperatures from Diss Mere and Meerfelder Maar exceed 15°C during the HTM, and there is no strong relationship between summer temperature and diversity at these sites. However, at Nautajärvi the average reconstructed temperatures are at this threshold which may explain both the greater average richness values (Section 4.1) and the improved correlation between temperature and richness. Nonetheless, as the correlation lacks significance, productivity, pH, soil development, vegetation change, and complexity of the aquatic community likely exert a stronger control than temperature alone (Velle et al. 2010; Engels et al. 2019). In support of this, the lake catchment of these sites during the mid‐Holocene would have been floristically rich, dominated by mixed deciduous woodland at the lower latitude sites (Peglar 1993; Litt et al. 2009) with boreal pine forests, alongside deciduous taxa, dominating at higher latitudes (Ojala and Alenius 2005). This would have contributed to the development of nutrient‐rich soils in the vicinity of the lakes impacting lacustrine productivity and the aquatic community. Over the mid‐Holocene, warm climates therefore buffered against wholesale changes to insect communities, offering a degree of community resilience.

As above, there are weak correlations between network skewness and summer temperature across the HTM. This, alongside continued negative skewness, suggests that at multi‐decadal timescales within the HTM, the chironomid assemblage did not undergo major structural change. This could be argued to reflect community resilience, but rapid fluctuations in network skewness in the mid‐Holocene from all three records do suggest changes in taxon connectivity. Doncaster et al. (2016) argue that changes in the number of rare taxa can be an indicator of environmental change, as rare taxa can fill niches created by specific environmental conditions. Increased abundance of rare taxa over the HTM (Appendix S1) at Diss Mere and Nautajärvi (and variability at Meerfelder Maar), alongside changes in network skewness, suggests approaching threshold changes (Carpenter and Brock 2006; Dakos et al. 2015) or a recovery post disturbance (Wang et al. 2024). Above (Section 4.1), it was argued that negative skewness could indicate a chironomid ecosystem under stress. Rapid shifts in skewness may therefore reflect a change in community resilience. Equally, negative skew associations could relate to non‐analog communities between the modern calibration set and the fossil record (Velle et al. 2010). As we see an increase in rare taxa at Diss Mere and Nautajärvi in the HTM, with slightly poorer fits to temperature and stronger non‐analog conditions (Figures S7 and S9) it is possible that rare taxa exert influence on the skewness data at this time (Mayfield et al. 2022).

### 4.3 Influence of Multi‐Decadal Cold Oscillations on Chironomid Diversity

Increasing data resolution reveals complex and variable Holocene climates (e.g., Mayewski et al. 2004; Wanner et al. 2014; Hernández et al. 2020; McKay et al. 2024; van Dijk et al. 2024). At multi‐decadal scales, climate is driven by high‐frequency variability including solar activity, volcanism, and internal variability within oceanographic or atmospheric circulation patterns (McKay et al. 2024). The recent detection of cooling at ca 5.9 ka BP represents a period of Little Ice Age‐like conditions in the mid‐Holocene (van Dijk et al. 2024) with additional cooling at 5.3 ka BP (e.g., Roland et al. 2015; van Dijk et al. 2024). These events are increasingly identified in climate models (e.g., MPI‐ESM, LOVECLIM) when the models are run with volcanic forcing enabled (Kobashi et al. 2017).

At Diss Mere, between 6.20 and 5.85 ka BP temperatures follow a downward trend; at Meerfelder Maar, two declines are identified over the same window but occur for single sample points only; and at Nautajärvi, temperatures reduce and then follow a rising trend between 6.31 and 5.94 ka varve years BP. It is therefore possible that the reductions in summer temperatures (~2°C) relate to those modeled temperature reductions at ca. 5.9 ka BP, albeit with a chronological offset at Nautajärvi, the reasons for which are unknown. At each site, changes in temperature associated with a later mid‐Holocene event are equivocal, with no obvious reconstructed cooling. Interestingly, it could be argued that the temperature reconstructions using the Norwegian calibration do show signs of this later event at Diss Mere and Nautajärvi, with clear declines in summer temperature between 5.3 and 5.0 ka BP (Figures S13 and S14). This highlights the importance of calibration set and model selection. Nevertheless, using the Norwegian‐Swiss model, we cannot identify a later mid‐Holocene climatic cooling with confidence.

Previous research has suggested that small temperature changes (< 2°C) result in minor changes to chironomid diversity (Engels et al. 2019). However, at the same time as cooling for the 5.9 ka BP event, at Diss Mere there are trends toward reduced taxonomic richness and network skewness (and a significant correlation between temperature and richness; *r* = 0.83, *p* = 0.011). At Nautajärvi, the trend in taxon richness is less clear, with slightly elevated turnover and a reduction in network skewness from 6.3 ka varve years BP, and at Meerfelder Maar, cooling coincides with increased turnover. It is postulated here that the small change in temperature drives changes in chironomid diversity. At Diss Mere, it is presumed that taxon loss and gain is driven by strongly connected taxa, as trends toward lower richness and decreased skewness match those from simulated data (e.g., Mayfield et al. 2022). The data suggest that *Abablesmyia* and *C. mancus‐*type are replaced by a complex assemblage including *Cladopelma*, *Tanytarsini*, and *P. bathophilla‐*type, which occupy similar littoral ecological niches. From Nautajärvi, small changes in turnover and skewness suggest taxon replacement, again by strongly connected taxa. Taxa with elevated abundance at Nautajärvi (*Corynoneura* and *Sergentia*) occupy both littoral and profundal habitat zones, further highlighting habitat diversity. Taking each site, it would suggest that during sustained warm periods, multi‐decadal cooling can influence chironomid diversity patterns, indicating added complexity and non‐linearity in the temperature–diversity relationship. Yet, while reductions in temperatures are shown to affect insect diversity, complex taxon assemblages suggest that the role of other forcing factors (e.g., hydrology, nutrient status, and oxygen conditions) cannot be discounted.

### 4.4 Impact of Temperature With Future Changes in Freshwater Biodiversity

We have shown that temperature, alongside other ecological factors, has a complex role in influencing aquatic insect diversity patterns in the Holocene. Future temperatures could increase by 1.2°C–3.4°C (SSP1‐2.6) or 4.1°C–8.5°C (SSP5‐8.5) over the next decades; alongside an increase in extreme events (heatwaves/droughts/extreme rainfall events) and changes to seasonal length (Collins et al. 2024). Such events will affect the thermal regime and hydrology of lakes, causing further stress to freshwater ecosystems. Where future temperatures approximate those of the HTM, we show minor diversity responses (to summer temperature directly). However, if temperatures exceed those of the HTM, impacts to freshwater biodiversity will likely be observed.

We suggest that at the highest latitudes, where temperatures will warm more quickly due to arctic amplification (Screen and Simmonds 2010), at the extreme range of projections (i.e., beyond HTM climates), cooler climate aquatic insects will disappear regionally as available habitat niches are diminished. We suggest this may occur at Nautajärvi, with localized extinction (e.g., of *M. insignilobus‐*type) and replacement by northerly migrating temperate taxa, increasing turnover and decreasing network skewness as the assemblage is under stress. We show that in the mid‐Holocene, where temperatures are up to 2°C warmer than present, at Diss Mere and Meerfelder Maar insect assemblages are dominated by generalist and temperate taxa. As climates warm beyond the range of mid‐Holocene values at lower latitudes, at slower rates (Previdi et al. 2021), the increase in mean annual surface and summer temperatures (Fischer and Schär 2009; Collins et al. 2024) will likely cause localized extinction of cool‐temperate taxa that may be at the edges of their thermal range (e.g., *Sergentia*, *Paratanytarsus*), with potential for range shifts of warm or sub‐tropical chironomids into these locations generating increased turnover. Declines in richness are also likely with extreme warming (comparable to that observed at the upper end of the temperature gradient; Engels et al. 2019), promoting taxon loss and insect‐poor assemblages.

Therefore, while regions most vulnerable to warming, such as the high latitudes, will likely experience biodiversity losses, some regions with less extreme warming might see diversity increase, potentially enhancing ecosystem resilience. However, range shifts of non‐native taxa could either enhance or reduce existing resilience, depending on whether they outcompete or fill critical roles within existing ecological structures (Chaffin et al. 2016). The influence of rising temperatures will not only affect chironomids but also have cascading effects on the rest of the ecosystem, negatively affecting food web structures and ecosystem connectivity (Pelletier et al. 2020), contributing to biodiversity loss. At the extreme range, thermal stress may limit biological functioning, leading to reduced ecosystem stability. Given we are potentially approaching multiple climatic tipping points (e.g., McKay et al. 2022) future changes to freshwater ecosystem resilience should be expected.

## 5 Conclusions

Under the pressure of future climate change, freshwater ecosystems face many challenges. This study demonstrates the complex relationship between summer temperature and aquatic insect diversity over the Holocene. We show that across a European transect, from a continental sub‐Arctic location to more maritime locations, differences in α‐diversity, β‐diversity, and network skewness can be observed in association with regional differences in summer temperature. However, at local site scales, habitat availability, macrophyte abundance, and oxygen conditions appear key in influencing diversity and structural metrics. For example, increased taxon richness and less negative network skewness coincide with cool summer temperatures at Nautajärvi. However, greater richness and skewness when viewed against the ecological traits of identified chironomids suggest increased habitat availability. While at Diss Mere and Meerfelder Maar, higher reconstructed temperatures and their influence on the lake ecosystem may work to reduce diversity. This research then broadly agrees with suggestions of a 15°C temperature threshold, beyond which chironomid diversity is affected, where we observe negative associations between summer temperature and diversity metrics (observed both in regional and local assessments).

A key component of this study was to disentangle the influence of low‐ and high‐frequency climate variability on aquatic insect diversity. We show that in the Holocene, at local low‐frequency scales, there is a limited relationship between summer temperature and aquatic insect diversity, with warm climates generally promoting assemblage stability. At local high‐frequency scales, across the HTM, we observe minimal changes to this relationship. While greater summer temperature variability, from high‐frequency analyses during the HTM, may have a small role in influencing chironomid diversity, alongside a shift to positive associations between temperature and turnover, habitat availability, lake trophic state, mixing regimes, and nutrient availability are considered more important. However, over a multi‐decadal cooling episode, a diversity response is observed, highlighting the complexity in the summer temperature and diversity relationship for aquatic insects.

Future warming beyond the range of the Holocene climates will increase threats to freshwater insect diversity. This is compounded by expected climate‐driven shifts in freshwater availability, environmental degradation, hydrological systems, the nutrient balance, and habitat loss, which we argue are important for maintaining aquatic insect diversity. We demonstrate that more work is needed to establish the total ecological and environmental response in different climate intervals. Diversity metrics should be reconstructed on other high‐frequency records in other time windows to establish whether the temperature–diversity relationship observed here is a product of sample resolution or warm periods generally. Furthermore, the reconstruction of additional climatic parameters (mean annual temperature, winter temperature, and precipitation) is urgently required to establish the link between past climate and ecosystem diversity outside of the instrumental period. We argue this is a principal research goal given expected future freshwater ecological and environmental stress.

## Author Contributions

**Ashley M. Abrook:** conceptualization, data curation, formal analysis, investigation, methodology, writing – original draft, writing – review and editing. **Peter G. Langdon:** conceptualization, methodology, writing – original draft, writing – review and editing. **Gordon N. Inglis:** conceptualization, writing – original draft, writing – review and editing. **Achim Brauer:** data curation, resources, writing – review and editing. **Paul Lincoln:** data curation, investigation, writing – review and editing. **Roseanna Mayfield:** software, writing – review and editing. **Antti E. K. Ojala:** data curation, resources, writing – review and editing. **Celia Martin‐Puertas:** data curation, funding acquisition, project administration, resources, writing – review and editing.

## Conflicts of Interest

The authors declare no conflicts of interest.

## Supporting information


Appendix S1

## Data Availability

The data that support the findings of this study are openly available at Zenodo at http://doi.org/10.5281/zenodo.15782051. Transient climate simulation data were obtained from the US National Science Foundation, National Centre for Atmospheric Research, data archive at http://doi.org/10.5065/CXB5‐TV56. Holocene temperature data using palaeoclimate data assimilation were obtained from Zenodo at https://doi.org/10.5281/zenodo.6426332. Varve thickness data from Diss Mere were obtained from Pangaea at https://doi.org/10.1594/PANGAEA.944411. The pollen‐based reconstruction of July temperature in Finland was obtained from Global and Planetary Change at https://doi.org/10.1016/j.gloplacha.2024.104462.
