# Supplementary material for: The Role of Summer Temperature on Aquatic Insect Diversity at Multi‐Decadal Scales Within the Holocene
Source: Glob Chang Biol. 2025 Aug 5;31(8):e70366. doi: 10.1111/gcb.70366 (PMC12323302; doi:10.1111/gcb.70366)
Supplement: Supplementary file 1 — Appendix S1 [file GCB-31-e70366-s001.pdf]

Supplementary Information for 'The role of summer temperature on aquatic insect diversity at multi-decadal scales within the Holocene'

Abrook. A.M., Langdon. P.G., Inglis. G.N., Brauer. A., Lincoln. P., Mayfield, R., Ojala. A.E.K. and Martin-Puertas. C

1.1 Chironomid abundance data

Across the three sites 129 chironomid taxa were identified in 92 samples analysed (including undifferentiated taxa). In total 82, 94 and 90 morphotypes were identified at Diss Mere, Nautajärvi and Meerfedler Maar, respectively (Figure S1, S2, S3), with 72 co-occurrences between Diss Mere and Meerfelder Maar, 59 co-occurrences between Nautajärvi and Diss Mere and 60 co-occurrences between Nautajärvi and Meerfelder Maar, demonstrating unique faunal assemblages at each lake site. Most of the different morphotypes encountered are aquatic with few terrestrial and semi-terrestrial species. The majority of taxa identified are warm or temperate morphotypes with those taxa aligned to colder climatic conditions identified within the Nautajärvi sequence (e.g. *Micropsectra insignilobus*-type, *Heterotrissocladius marcidus*-type and *Synorthocladius*) as defined by the weighted average species thermal optima. Across the three sites five assemblage zones were identified throughout the Diss Mere sequence (Figure S1), and four at both Nautajärvi (Figure S2) and Meerfelder Maar (Figure S3) respectively. Zone and chironomid taxon info are provided in Table 1 within the main body of the manuscript.

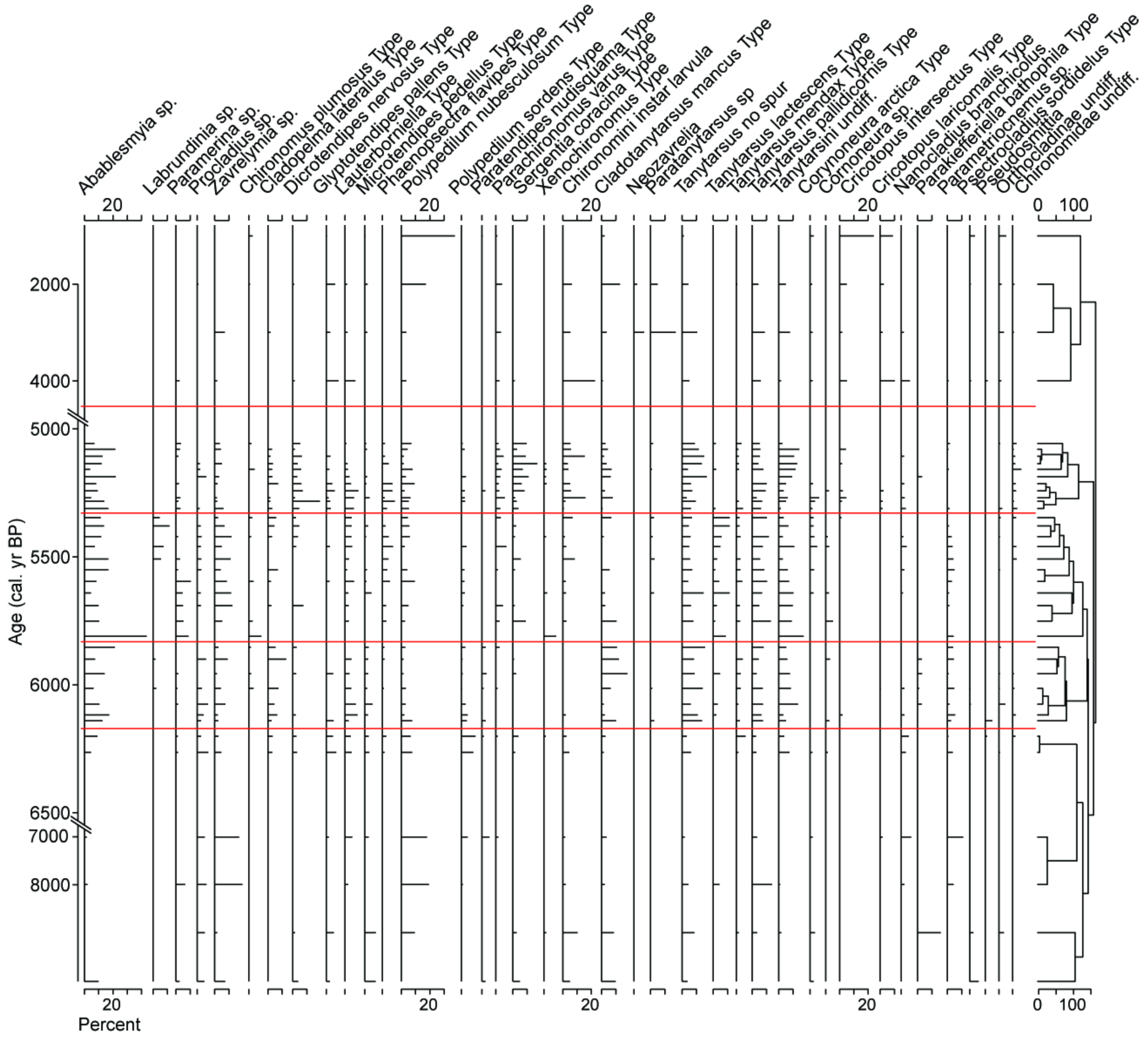

Figure S1. Chironomid assemblage diagram from Diss Mere. Shown are identified chironomid head-capsules >5% abundance. To highlight the higher resolution mid-Holocene, the age scale has been expanded between 6.5 and 5 ka BP. Red bars denote the chironomid zones and a dendrogram highlights the major zones in the assemblage.

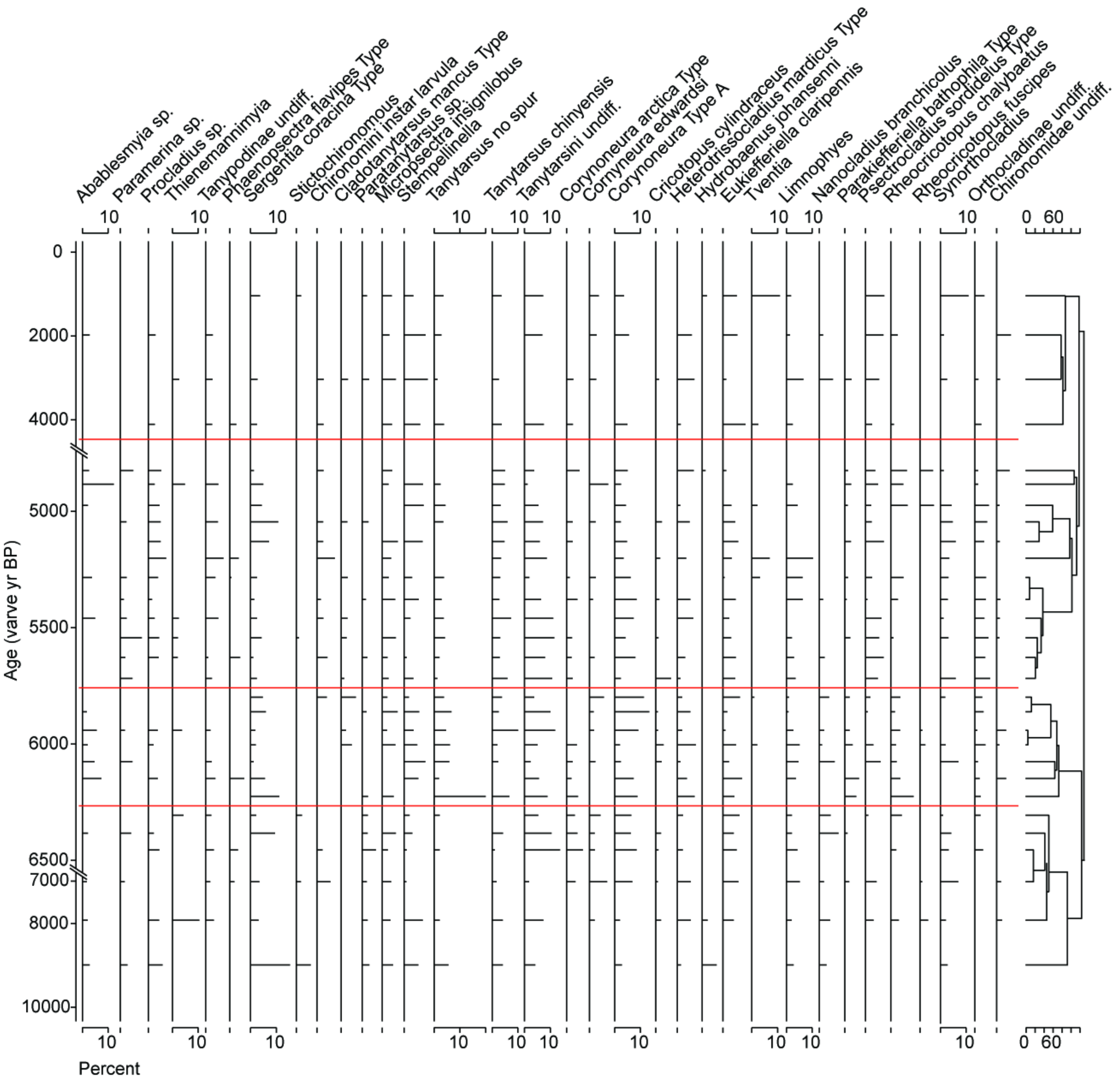

Figure S2. Chironomid assemblage diagram from Nautajärvi. Shown are identified chironomid head-capsules >5% abundance. To highlight the higher resolution mid-Holocene, the age scale has been expanded between 6.5 and 4.7 ka BP. Red bars denote the chironomid zones and a dendrogram highlights the major zones in the assemblage.

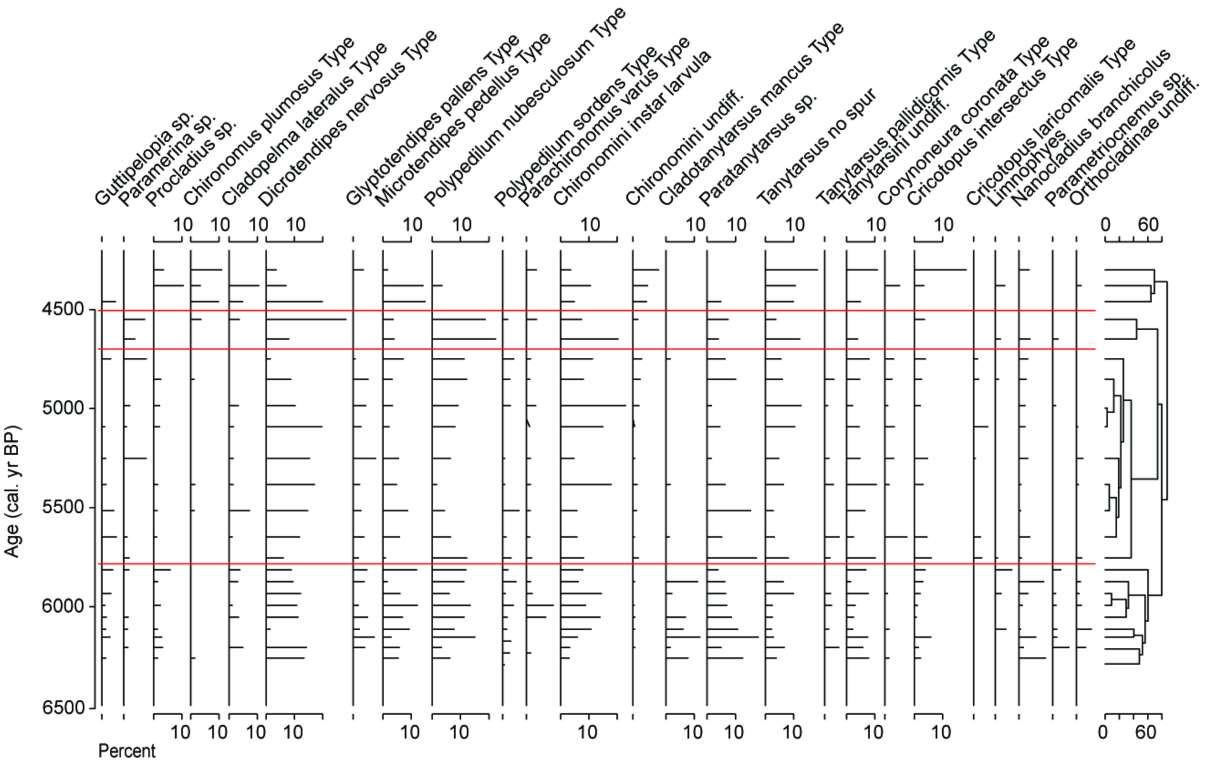

Figure S3. Chironomid assemblage diagram from Meerfelder Maar. Shown are identified chironomid head-capsules >5% abundance. Note this diagram contains the mid-Holocene only. Red bars denote the chironomid zones based on the dendrogram.

Table S1. Samples where head capsule counts fall below the cut off of 40 head capsules.

	Diss Mere		Nautajärvi		Meerfelder Maar	
**Sample**	**Age**	**HC number**	**Age**	**HC number**	**Age**	**HC number**
**1**	5187	32	1042	27.5	4231	27
**2**	5509	36	1975	36	4333	28
**3**	5751	38.5	3029	38	4436	20
**4**	5811	11	4824	39.5	4539	26.5
**5**	5853	37.5	5201	29	4642	24.5
**6**	6141	39.5	6148	27	6141	33
**7**			6225	22.5		
**8**			8990	36		
**9**			9821	1		

Table S2. Head capsule counts of samples after taxon rounding to whole integers for rarefaction analysis (following the method of Engels et al. 2019). All samples whose HC counts are ≥ 40 are included in correlations.

	Diss Mere		Nautajärvi		Meerfelder Maar	
**Sample**	**Age**	**HC number**	**Age**	**HC number**	**Age**	**HC number**
**1**	5187	34	1042	31	4231	28
**2**	5509	37	5201	31	4333	30
**3**	5811	12	6148	32	4436	20
**4**			6225	27	4539	28
**5**			9821	2	4642	26
					6141	35

1.2 Chironomid Sensitivity Testing

To test the reliability of each reconstruction, a series of sensitivity tests were performed using different groupings of the fossil data alongside the calibration sets. Three separate sensitivity tests were performed at each site for the Norwegian-Swiss model whilst two tests were performed for the Norwegian model. The reason for performing these tests was to establish whether different groupings of taxa affect individual reconstructions and to test the effect of low head-capsule sums (<40 or after discarding those that do not exist in training sets) on reconstructions. These tests therefore inform whether there is any bias in the reconstruction if morphotypes not present within the modern calibration set are excluded or merged within lower taxonomic classifications or with similar morphotypes (a process common in palaeoecological reconstruction).

For the Nor-Swiss model the fossil datasets were either 'Ungrouped', '*Tanytarsini* no-spur and *Paratanytarsus* Grouped' or 'Completely Grouped'. The 'Ungrouped' category removed all taxa not identified within the training set from the analysis (except those that are normally combined e.g. *C. laricomalis*-type and *C. intersectus*-type as Cric 292). The middle reconstruction of '*T.* no-spur and *Paratanytarsus* Grouped' involved the creation of a *Paratanytarsus* undiff. group in the training set and grouping of all fossil *Paratanytarsus* morphotypes due to the lack of preserved mandibles. This second reconstruction also split all recorded *Tanytarsini* no-spur across *Tantarsus mendax*-type and *Tanytarsus nemorous*-type, again due to the lack of mandibles preserved. All other morphotypes not identified within the modern training sets were excluded from the reconstruction. The final reconstruction, 'Grouped', involved grouping as many identified fossil taxa not present in the modern dataset into lower taxonomic classifications or morphotypes that are present. This included proportionally splitting *Corynoneura* sp across identified *Corynoneura* morphotypes and *Tanytarsini* undiff across identified *Tanytarsini* groups. This also included adding taxa like *Parakiefferiella* sp and *Psectrocladius* sp. into *p. bathophilla*-type and *p. sordidellus*-type respectively.

For the Norwegian model the datasets were either ‘Ungrouped’ or ‘Grouped’. The approach followed the same process as the Norwegian-Swiss model with the ‘Ungrouped’ data leaving out all fossil taxa from the reconstruction that are not seen within the modern dataset. The ‘Grouped’ approach grouped those taxa not identified within the modern dataset into suitable morphotypes. This is less of a concern with the Norwegian dataset, owing to the broader range of undifferentiated categories in the modern dataset.

From this data, for the most part, grouping the different morphotypes makes very little difference to any reconstruction, both within the Norwegian-Swiss and the Norwegian models. There is mostly a strong degree of overlap within each reconstruction and the trends within each reconstruction remain. Where there are differences includes the upper samples within the Ungrouped combined reconstruction and the ungrouped reconstruction at Meerfelder Maar. For the latter this can be explained by the identification of *Paratanytarsus* in the fossil data. As ‘ungrouped’ these are left out of the analysis. At Meerfelder Maar *Paratanytarsus* was a major component of the mid-Holocene assemblage. Excluding these likely impacted reconstructions. Therefore, whilst the progressive inclusion of more taxa (owing to taxonomic grouping) circumvents potential issues around lower head-capsule numbers, the reconstructions show little variability when compared to those with lower head-capsule abundances. So it makes little difference which reconstruction is selected. Nonetheless for the reconstructions within the main body of the manuscript we present the second reconstruction of the Norwegian-Swiss model (T. no spur and *Paratanytarsus*) and the Ungrouped reconstruction in the Norwegian model.

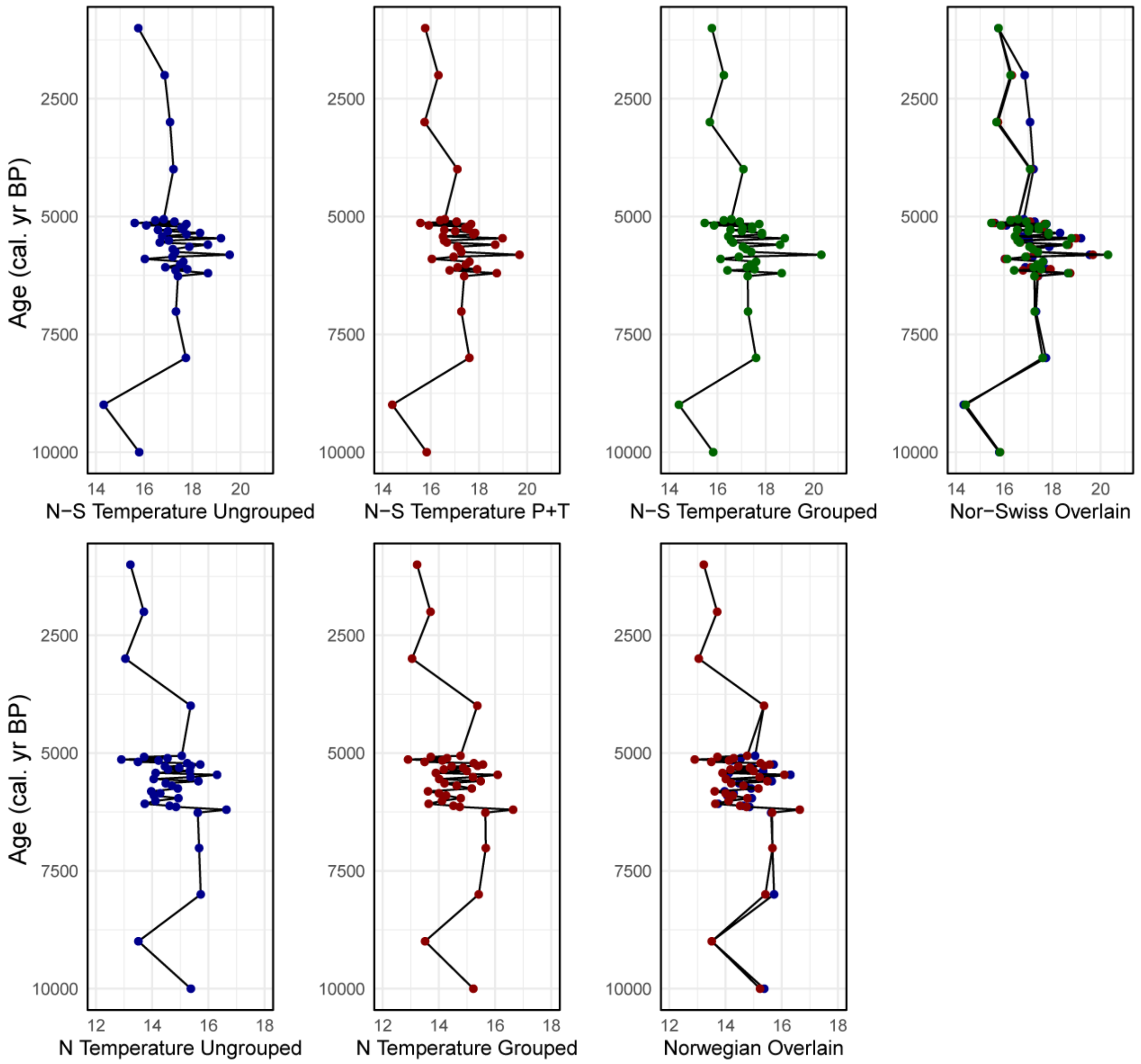

Figure S4. Highlighting the different chironomid-based temperature reconstructions from Diss Mere across the Holocene. The broad comparability between reconstructions are shown in the overlain plots. N-S are the Norwegian-Swiss models; N is the Norwegian Model. Ungrouped, P+T and Grouped relates to differences in the grouping of fossil chironomid taxa as displayed in text.

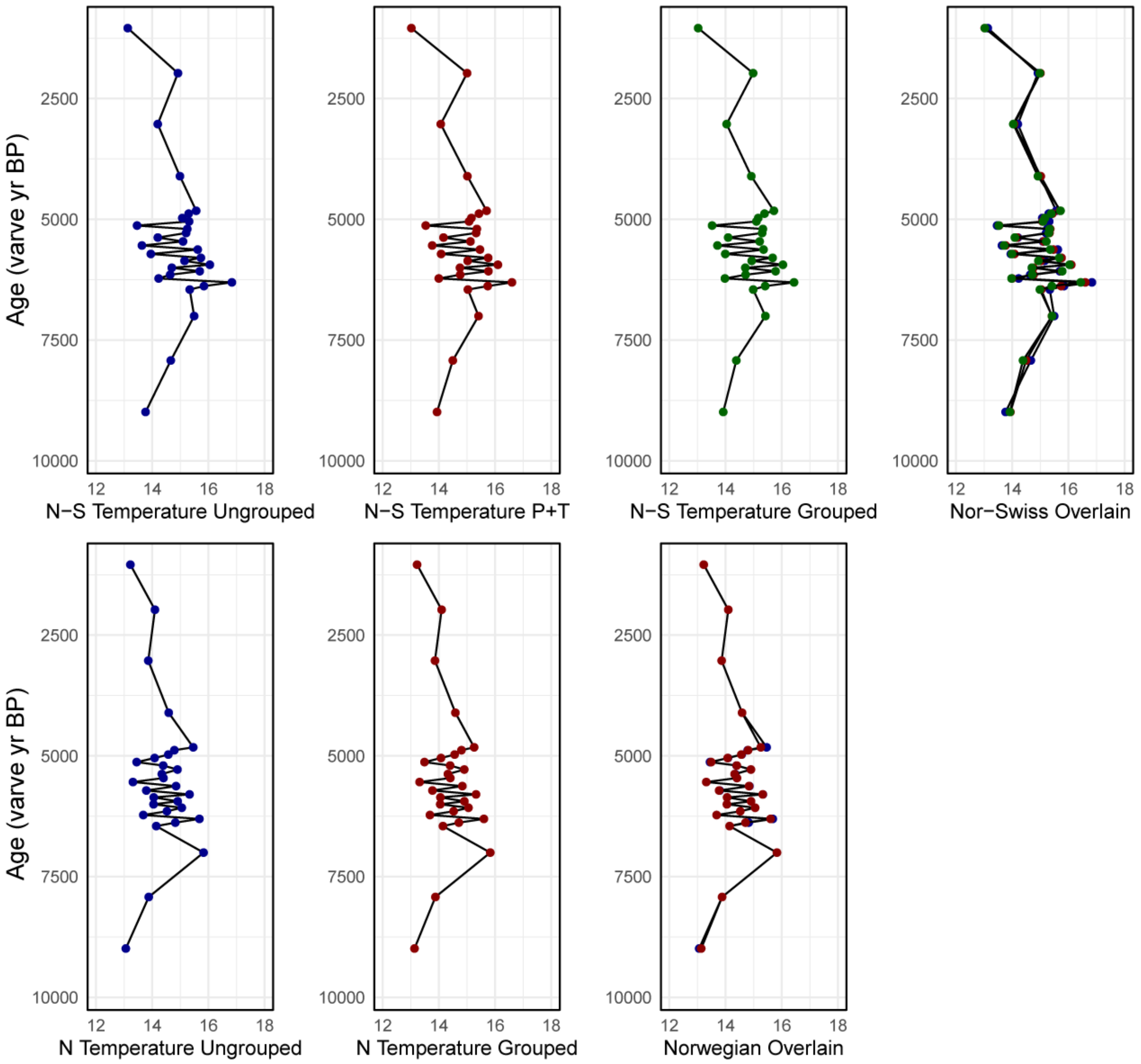

Figure S5. Highlighting the different chironomid-based temperature reconstructions from Nautajärvi across the Holocene. The broad comparability between reconstructions are shown in the overlain plots. At Nautajärvi the difference between the Norwegian and Swiss reconstructions are minimal. N-S are the Norwegian-Swiss models; N is the Norwegian Model. Ungrouped, P+T and Grouped relates to differences in the grouping of fossil chironomid taxa as displayed in text.

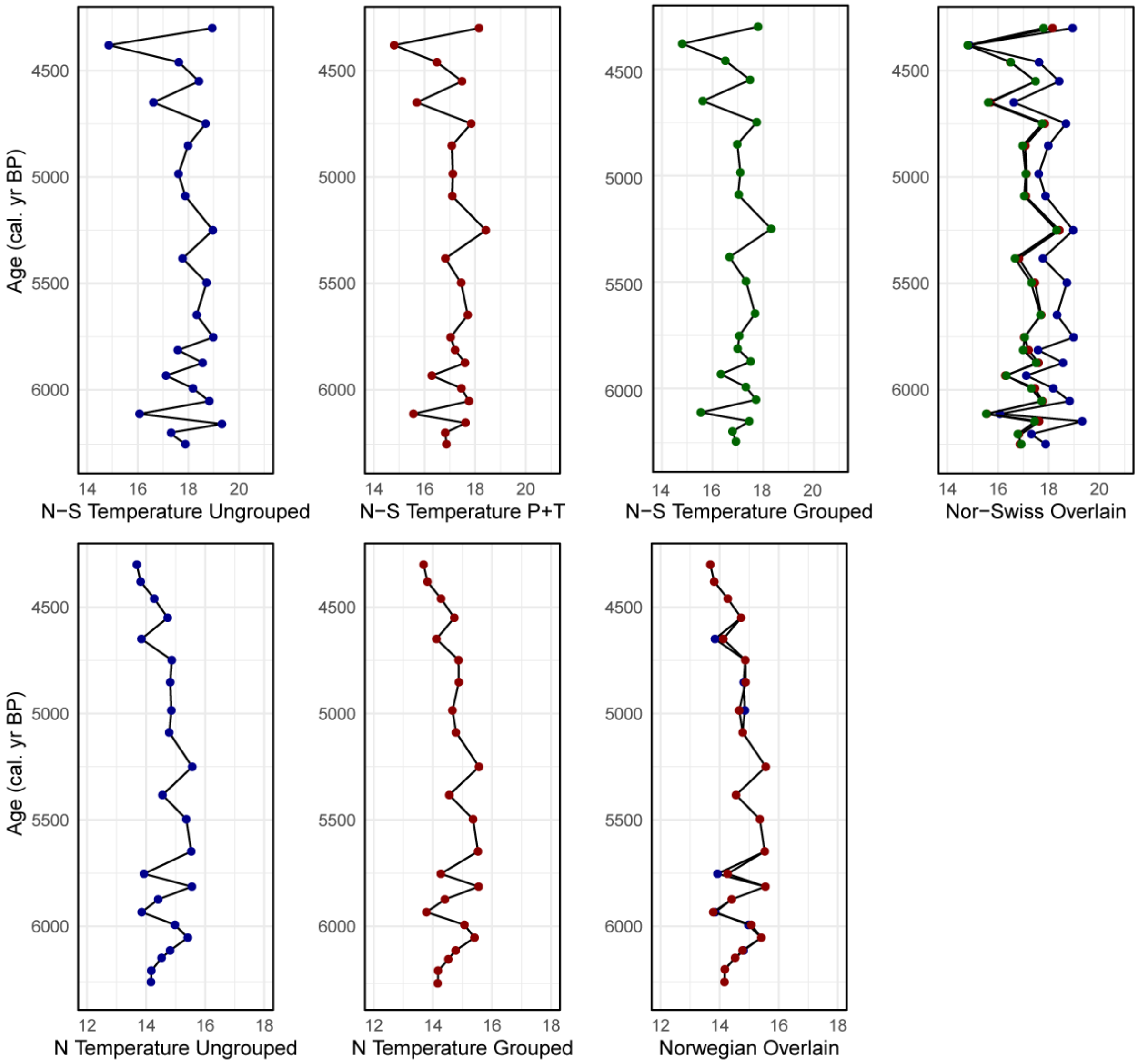

Figure S6. Highlighting the different chironomid-based temperature reconstructions from Meerfelder Maar across the mid-Holocene. The broad comparability of the temperature reconstructions are shown in the overlain plots. At Meerfelder Maar the difference between the N-S Temperature Ungrouped and N-S Temperature P+T/Grouped is clear. N-S are the Norwegian-Swiss models; N is the Norwegian Model. Ungrouped, P+T and Grouped relates to differences in the grouping of fossil chironomid taxa as displayed in text.

.

1.3 Chironomid Reconstruction Diagnostics

Alongside the chironomid sensitivity tests, a series of diagnostic tests were performed on the final chironomid reconstructions, using the second (T. no spur and *Paratanytarsus* grouping) reconstruction of the Norwegian-Swiss model and the first reconstruction of the Norwegian model (see Section 1.1). These diagnostic tests are a further way to test the robustness of the reconstructions. For goodness of fit to temperature, tests were performed using Canonical Correspondence Analysis (CCA) with the modern percentage data and mean July temperature as the only constraining variable. Residual lengths of the fossil dataset were estimated by passively plotting the fossil percentage data into the CCA and comparisons were made between the residual lengths of both the calibration data and fossil data to the first CCA axis (i.e the July temperature constrained axis; axis 1). 90th and 95th percentiles were extracted from all residuals within the calibration dataset and where fossil samples fall beyond these percentiles, the samples are assumed to have a ‘poor’ and ‘very poor’ fit to temperature. Goodness of fit to temperature analyses were performed using the ‘Vegan’ and ‘Analogue’ packages in R (Oksanen et al., 2024; Simpson et al., 2024) using square-root transformed percentage data. Second, as an assessment of dissimilarity, samples were assessed to detect whether the modern calibration set are good analogues for the fossil data. Here the calibration and fossil percentages were proportioned, and dissimilarity indexes were calculated between the modern calibration data and the fossil data. The distance metric used here is the chi. square distance (Simpson, 2007) with minimum distances between the modern and fossil data returned and plotted stratigraphically. Dissimilarity cut-off values of 5th and 10th percentiles of all chi. square distances in the modern calibration set were used to define whether fossil samples beyond these percentiles had ‘no close’ or ‘no good’ analogues respectively (Simpson, 2007). Analogue and dissimilarity approaches were conducted using the ‘Analogue’ package in R (Simpson et al., 2024). As a final check on model applicability, assessments of taxon rarity and absence have been produced. For rarity, at each site, cumulative percentages of fossil taxa were summed where those taxa in the modern assemblage data had a Hill’s N2

of <5; and for absence, a summation of the percentages of all fossil taxa which are not present in the different calibration sets. These data are shown in supplementary figures 7, 9, 11 (for the combined Norwegian-Swiss model at Diss Mere, Nautajärvi and Meerfelder Maar respectively) and supplementary figures 8, 10, 12 for the Norwegian model.

For fit to temperature both Diss Mere and Meerfelder Maar have mostly a good fit to temperature using the Norwegian-Swiss data. The fit to temperature is considerably poorer when just using the Norwegian data with more sample horizons displaying a poor and very poor fit to temperature. At Nautajärvi, both the Norwegian-Swiss and Norwegian calibrations produce poorer fits to temperature than the two former sites with a larger proportion of no good fit to temperature. It is interesting that across the three sites the samples with the poorest fit to temperature occur in the mid-Holocene. Barring a samples from Nautajärvi at 9.8 ka BP- although this is an erroneous sample as it contains very few head-capsules. The fact that the fit to temperature is poor for specific samples in the mid-Holocene is interesting and that perhaps, there may be other controls on the fossil chironomid data in the mid-Holocene that just July temperature alone. This is not thought to be problematic at the lower latitude sites as the fit to temperature is mostly good throughout.

With the modern analogue assessments it is clear that better analogues are produced when using the Norwegian-Swiss model than just using the Norwegian model alone, for each site. Much like the fit to temperature there are a few datapoints that demonstrate no good analogue (>10th percentile) or no close analogues (>5th percentile) across each of the sites when distances are compared to the combined modern dataset. However these are far fewer than the Norwegian data where, for example, at Meerfelder Maar, across the mid-Holocene no sample has good analogues and at Nautajärvi and Diss Mere, where all samples either fall into no close or no good analogues. This suggests that for Diss Mere, Nautajärvi and for Meerfelder Maar the Norwegian calibration model is much poorer in terms of analogue quality and that there is a disparity between the fossil and Norwegian calibration set assemblages. This is perhaps not surprising for Diss Mere and Meerfelder Maar where the two sites are very

different in terms of their latitudinal and continental positions to Norway, and that the former lake exhibits carbonate production in the summer hence a better fit to the Norwegian-Swiss dataset. It is a little more surprising for Nautajärvi given proximity to Norway. However, Nautajärvi is a slightly more acidic system so perhaps that is a reason for the dissimilarity.

In terms of rarity and absence, there are fewer rare taxa identified using the merged Norwegian-Swiss data set across all sites, barring between 5.0-6.0 ka BP where rare taxa increase at Diss Mere. However, rare taxon percentges are also relatively low from Nautajärvi and Meerfelder Maar. In terms of taxon absence, there are far more identified fossil taxa that are absent from the merged Norwegian-Swiss calibration set than the Norwegian dataset. Absent taxa are quite low when compared to the Norwegian dataset- apart from 9.8 ka BP which as already established is based on low head-capsule abundances.

Therefore taken together, goodness of fit, analogue quality and an assessment of taxon rarity and absence, the most suitable reconstructions for each site are those based on the Norwegian-Swiss model. Whilst taxa not present within the training set are higher across all sites, the fit to temperature, analogue quality and rarity of taxa are all stronger using the combined Norwegian-Swiss approach. This therefore is the main reason for prioritising this model in the main body of the manuscript. Whilst taxon absence may present a concern, the sensitivity tests (Supplementary 1.1), where we have grouped as many taxa as possible to reduce the total number of absent taxa across each reconstruction, demonstrates that our reconstructions are reproducible regardless of the number of absent taxa and those contained low head-capsule numbers. The Norwegian-Swiss reconstruction for each site is therefore robust.

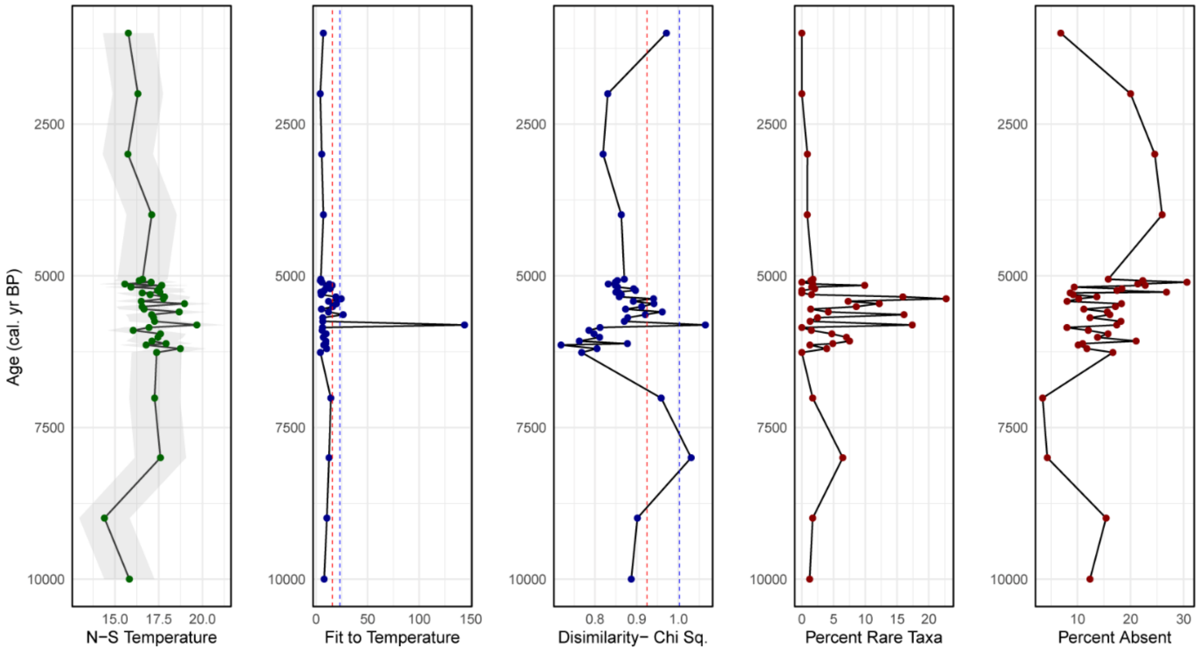

Figure S7. Showing the Norwegian-Swiss reconstruction and reconstruction diagnostics for the combined model at Diss Mere. From left to right panels show fit to temperature using residual lengths of fossil and modern reconstructions (red and blue dashed bars display the 90th and 95th percentile of the modern dataset respectively), modern analogue approaches using distance-based metrics (red and blue dashed bars demonstrate the 5th and 10th percentiles of the modern dataset). Also shown are the percentage of rare taxa in fossil samples as derived from the modern dataset and total number of absent taxa when comparing the fossil and modern calibration sets.

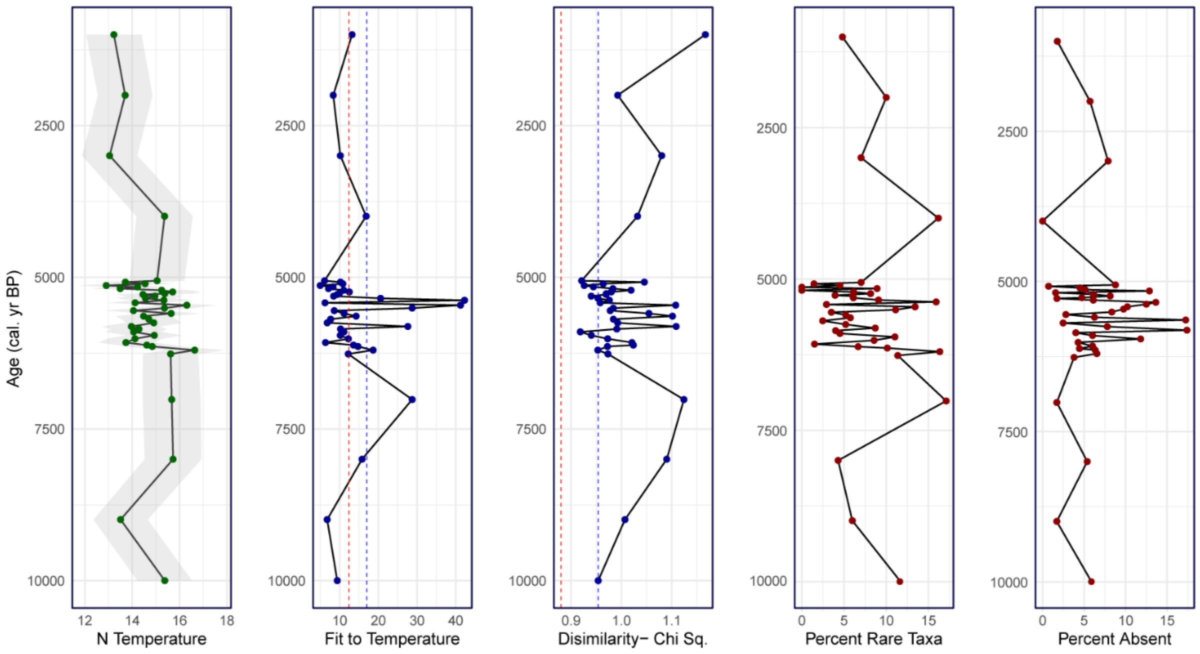

Figure S8. Showing the Norwegian reconstruction and reconstruction diagnostics for the Norwegian model at Diss Mere. From left to right panels show fit to temperature using residual lengths of fossil and modern reconstructions (red and blue dashed bars display the 90th and 95th percentile of the modern dataset respectively), modern analogue approaches using distance-based metrics (red and blue dashed bars demonstrate the 5th and 10th percentiles of the modern dataset). Also shown are the percentage of rare taxa in fossil samples as derived from the modern dataset and total number of absent taxa when comparing the fossil and modern calibration sets.

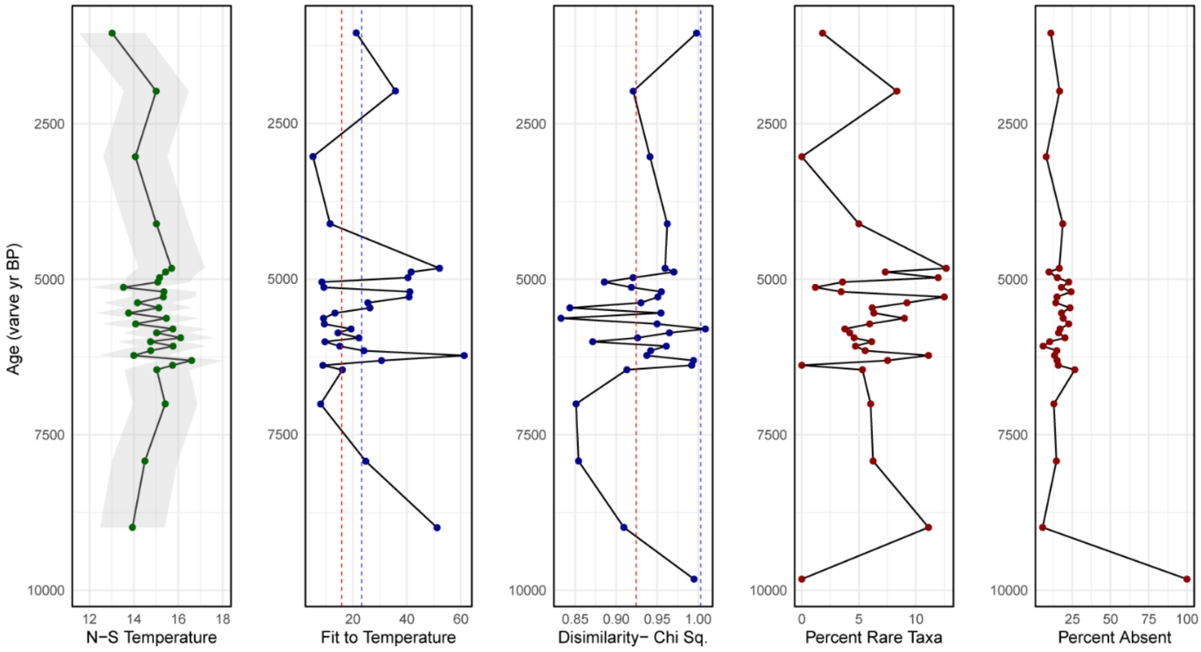

Figure S9. Showing the Norwegian-Swiss reconstruction and reconstruction diagnostics for the combined model at Nautajärvi. From left to right panels show fit to temperature using residual lengths of fossil and modern reconstructions (red and blue dashed bars display the 90th and 95th percentile of the modern dataset respectively), modern analogue approaches using distance-based metrics (red and blue dashed bars demonstrate the 5th and 10th percentiles of the modern dataset). Also shown are the percentage of rare taxa in fossil samples as derived from the modern dataset and total number of absent taxa when comparing the fossil and modern calibration sets.

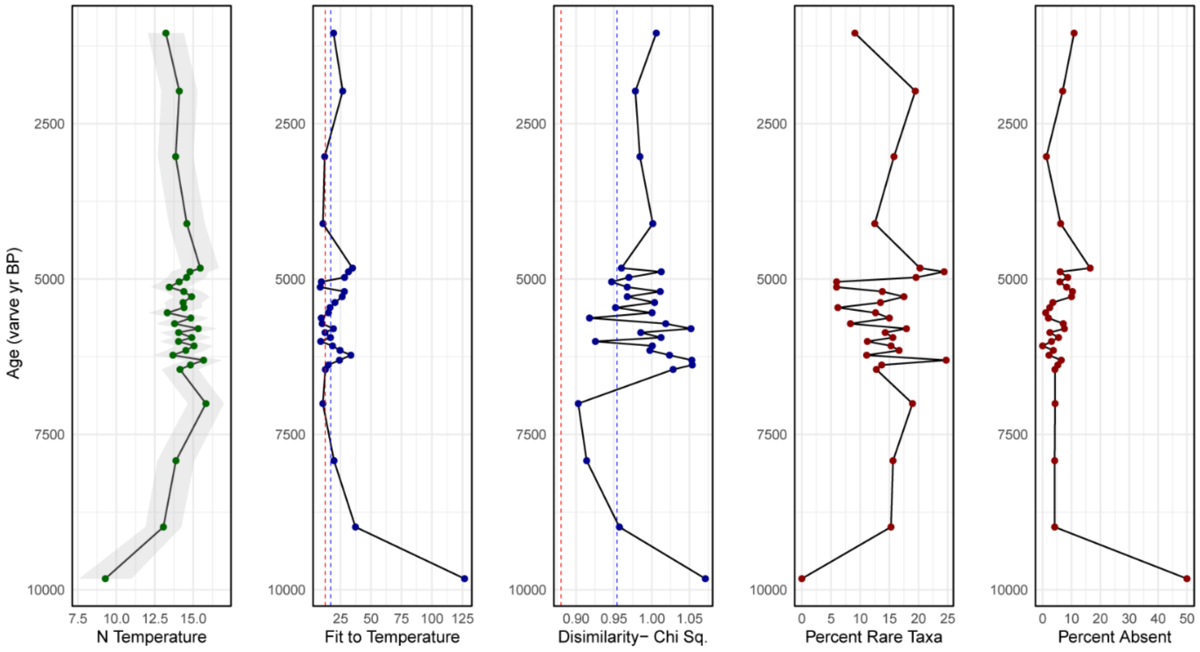

Figure S10. Showing the Norwegian reconstruction and reconstruction diagnostics for the Norwegian model at Nautajärvi. From left to right panels show fit to temperature using residual lengths of fossil and modern reconstructions (red and blue dashed bars display the 90th and 95th percentile of the modern dataset respectively), modern analogue approaches using distance-based metrics (red and blue dashed bars demonstrate the 5th and 10th percentiles of the modern dataset). Also shown are the percentage of rare taxa in fossil samples as derived from the modern dataset and total number of absent taxa when comparing the fossil and modern calibration sets.

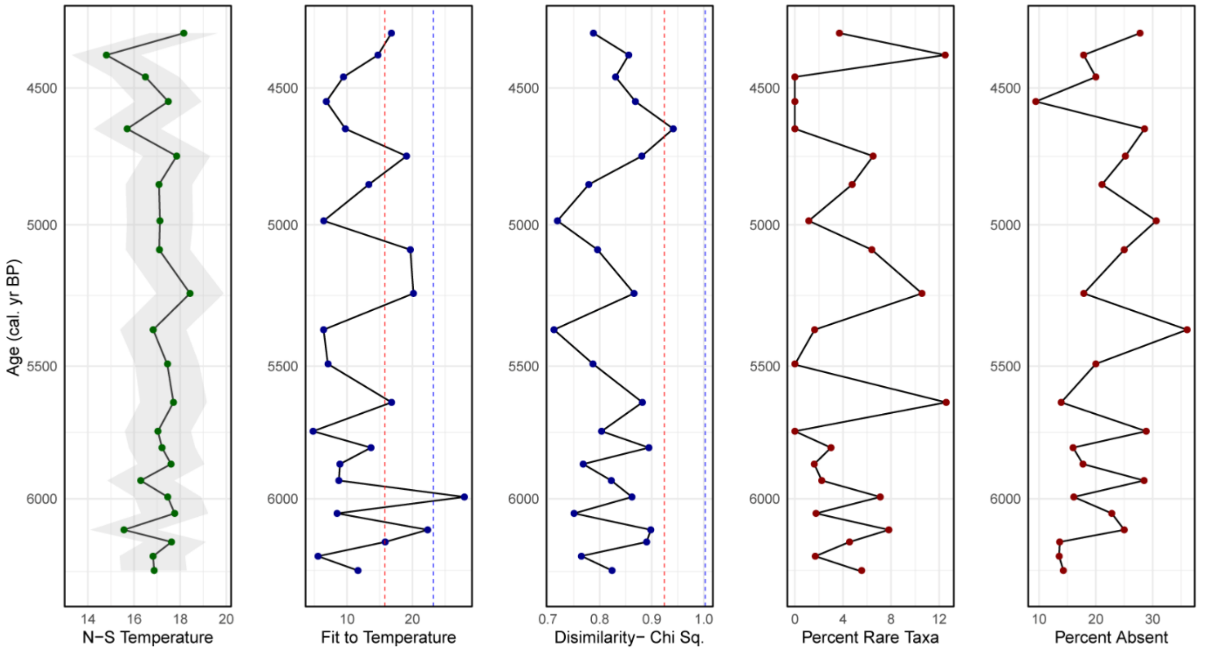

Figure S11. Showing the Norwegian-Swiss reconstruction and reconstruction diagnostics for the combined model at Meerfelder Maar. From left to right panels show fit to temperature using residual lengths of fossil and modern reconstructions (red and blue dashed bars display the 90th and 95th percentile of the modern dataset respectively), modern analogue approaches using distance-based metrics (red and blue dashed bars demonstrate the 5th and 10th percentiles of the modern dataset). Also shown are the percentage of rare taxa in fossil samples as derived from the modern dataset and total number of absent taxa when comparing the fossil and modern calibration sets.

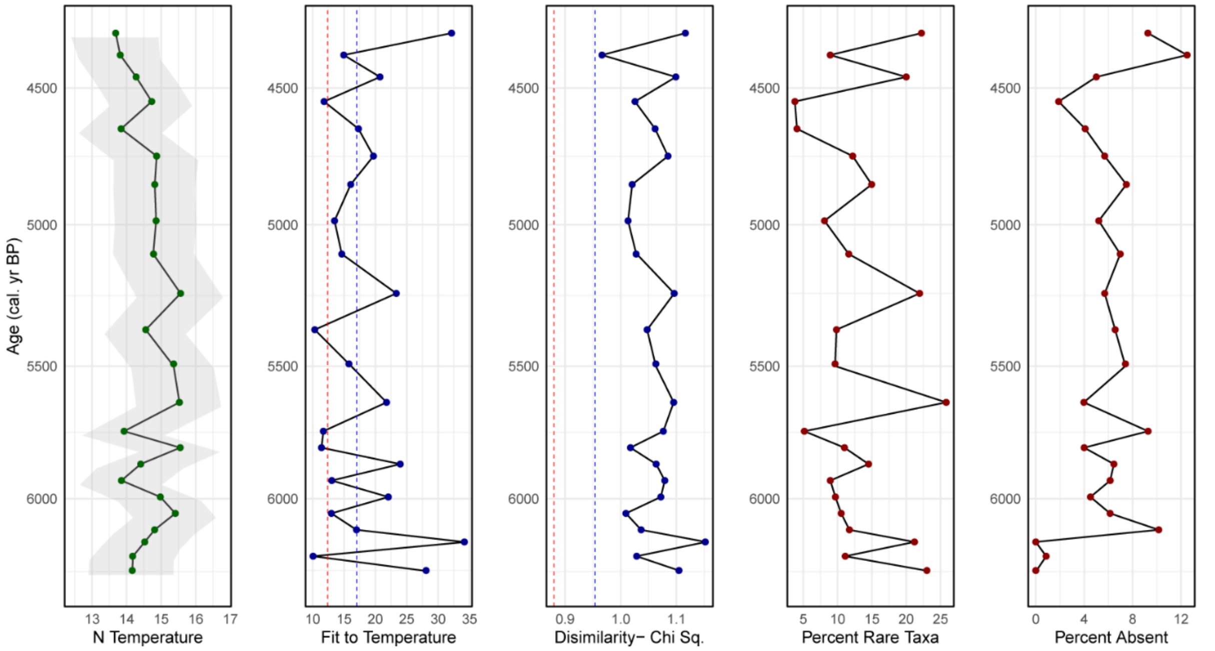

Figure S12. Showing the Norwegian reconstruction and reconstruction diagnostics for the Norwegian model at Meerfelder Maar. From left to right panels show fit to temperature using residual lengths of fossil and modern reconstructions (red and blue dashed bars display the 90th and 95th percentile of the modern dataset respectively), modern analogue approaches using distance-based metrics (red and blue dashed bars demonstrate the 5th and 10th percentiles of the modern dataset). Also shown are the percentage of rare taxa in fossil samples as derived from the modern dataset and total number of absent taxa when comparing the fossil and modern calibration sets.

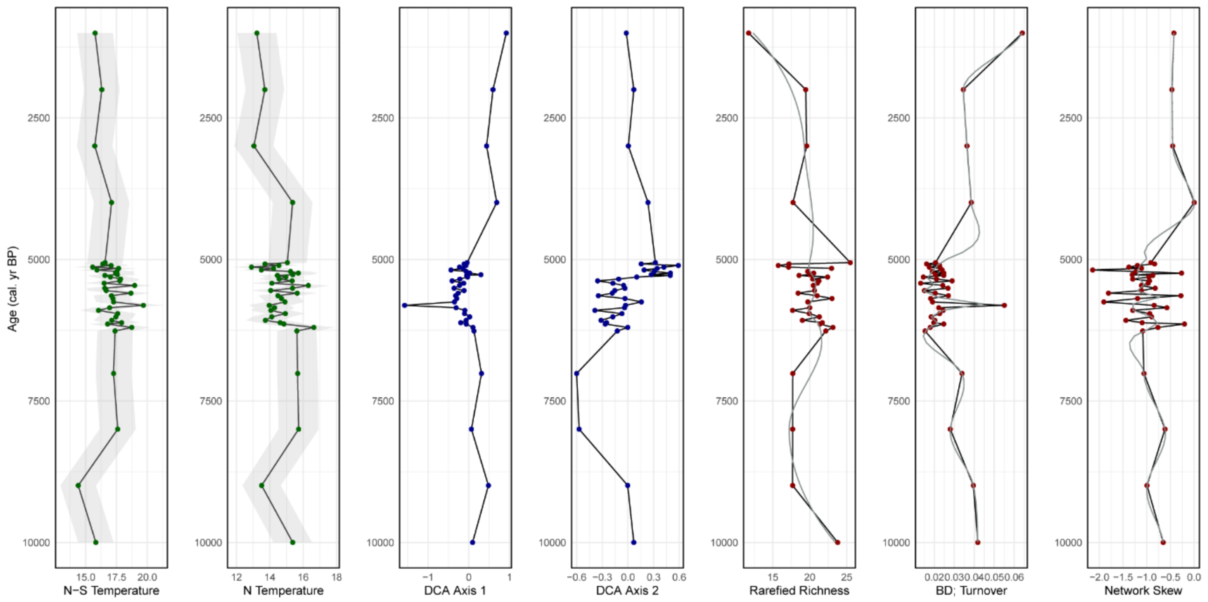

Figure S13. Summer temperature and diversity reconstructions from Diss Mere. From left to right shown are summer temperature reconstructions based on 1) a Norwegian-Swiss temperature model; 2) a Norwegian temperature model, both models display sample specific errors; detrended correspondence analyses axis-1 and axis-2 scores; rarefied richness scores indicating α-diversity; turnover scores indicating β-diversity and network skew indicating connectivity. Added to the diversity metrics are LOESS smoothers with variable span.

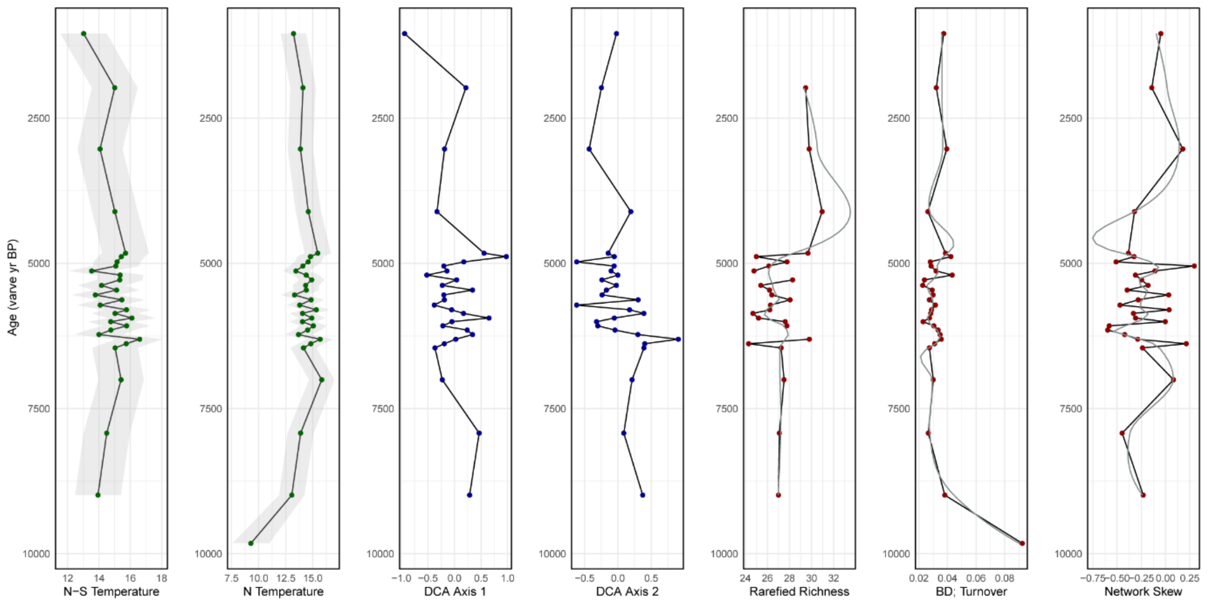

Figure S14. Summer temperature and diversity reconstructions from Nautajärvi. From left to right shown are summer temperature reconstructions based on 1) a Norwegian-Swiss temperature model; 2) a Norwegian temperature model, both models display sample specific errors; detrended correspondence analyses axis-1 and axis-2 scores; rarefied richness scores indicating α-diversity; turnover scores indicating β-diversity and network skew indicating connectivity. Added to the diversity metrics are LOESS smoothers with variable span.

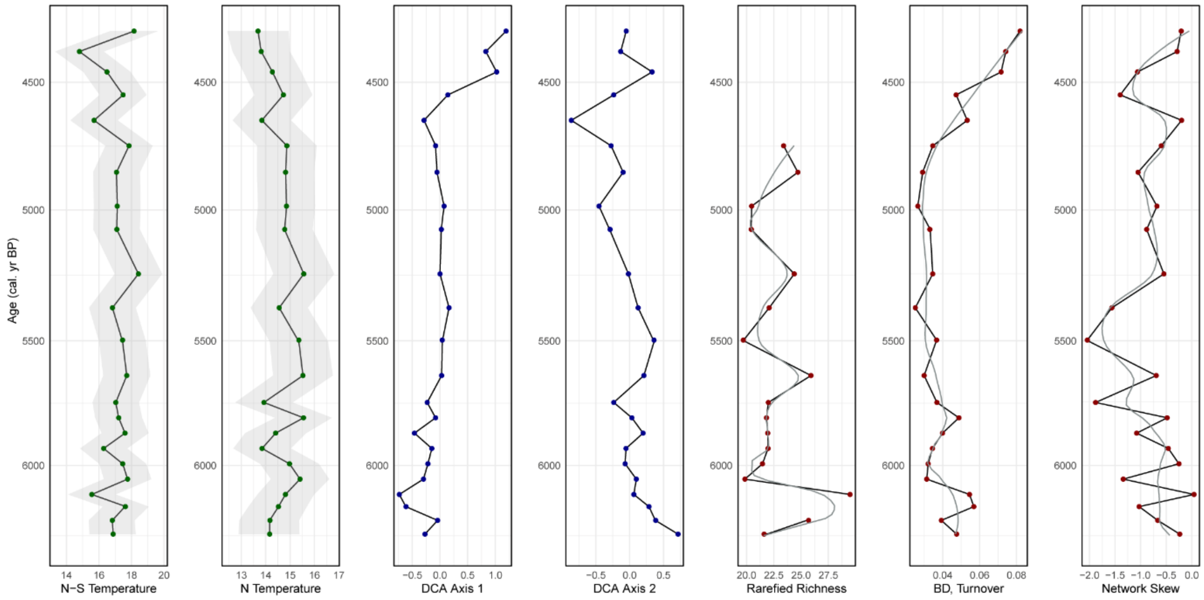

Figure S15. Summer temperature and diversity reconstructions from Meerfelder Maar. From left to right shown are summer temperature reconstructions based on 1) a Norwegian-Swiss temperature model; 2) a Norwegian temperature model, both models display sample specific errors; detrended correspondence analyses axis-1 and axis-2 scores; rarefied richness scores indicating α-diversity; turnover scores indicating β-diversity and network skew indicating connectivity. Added to the diversity metrics are LOESS smoothers with variable span.

1.4 Temperature – Diversity Correlations

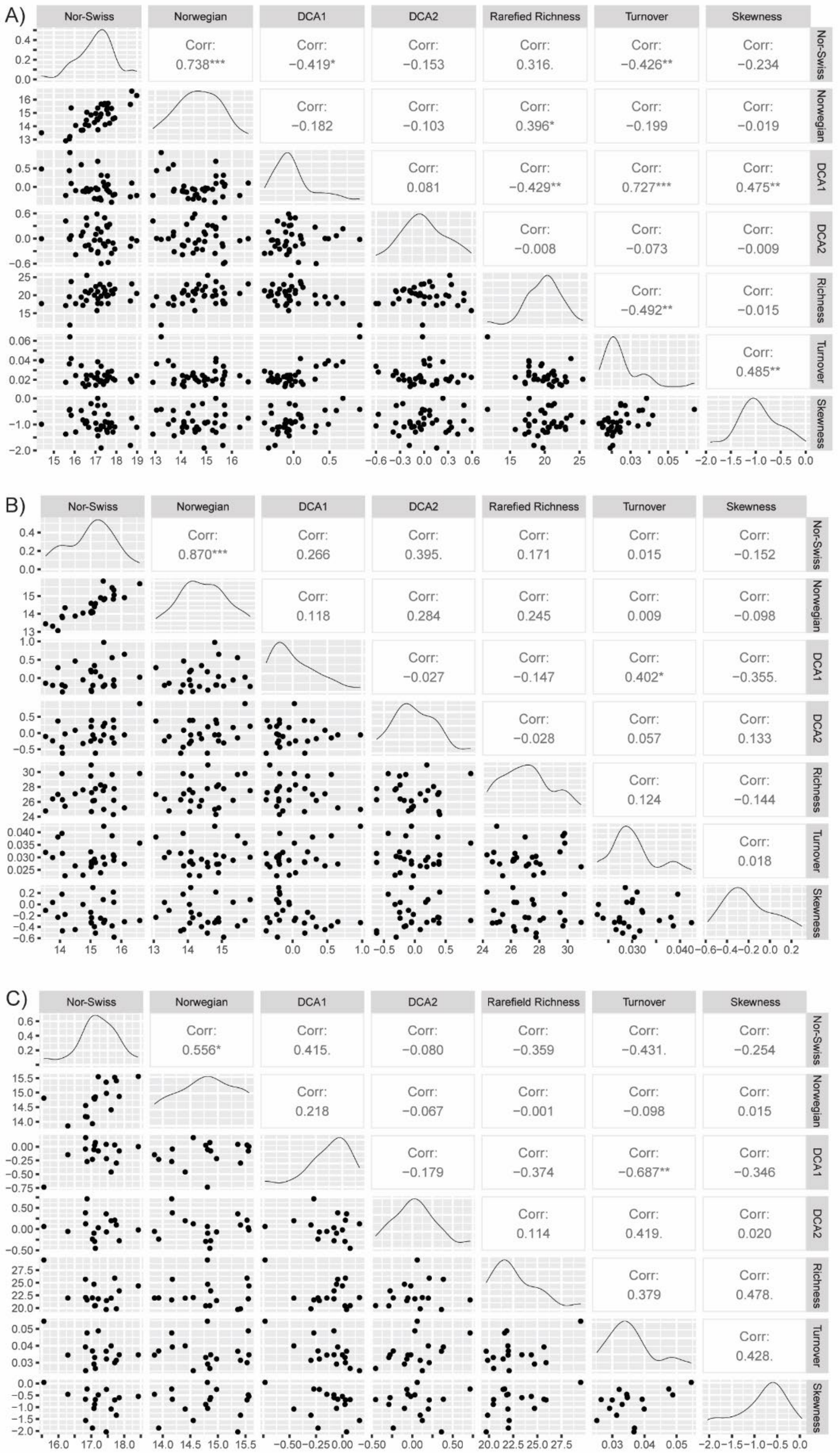

Figure S16. Pearsons correlation coefficients for the different reconstructions and diversity calculations from Diss Mere (A); Nautajärvi (B); and Meerfelder Maar (C). These correlations are performed across the whole dataset from each site.

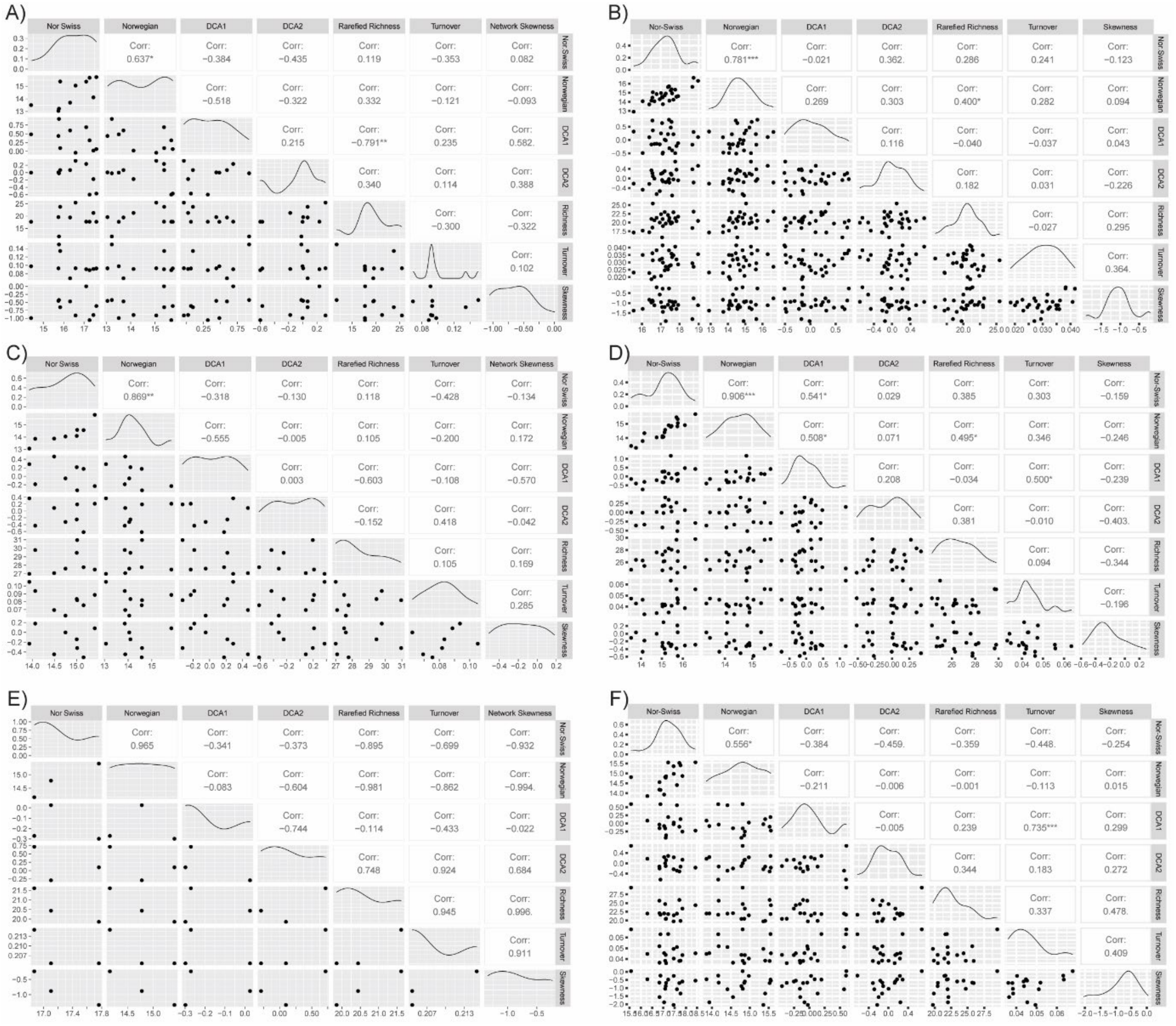

Figure S17. Showing the Pearsons correlation coefficients for the reconstructions and diversity calculations from Diss Mere (A, B); Nautajärvi (C, D); and Meerfelder Maar (E, F). For each horizontal panel, displayed first are the low-frequency trends (A, C, E) and then the mid-Holocene high-frequency trends (B, D, F). There are very few correlative points in E) owing to fewer samples counted for chironomids. This panel is included for completeness only. It is not referred to in the main body of the text.

1.5 Within lake and regional data comparisons

The data produced by this study in terms of the chironomid-inferred summer temperature reconstructions is comparable to independent data from each lake site, or to regional reconstructions using data assimilation approaches or transient simulations. We show that the Diss Mere reconstructions (Figure S18) broadly show warmest temperatures during the mid-Holocene which aligns with regional data-assimilation approaches over the same latitudinal band (Erb et al., 2022). Perhaps most clearly the data is strongly aligned to the In(Ca/Ti) ratio which has been interpreted as the element ratio that explains greater calcite production in the lake versus detrital (titanium) input (i.e. a summer signal; Boyall et al., 2024). Here values increase from 8 ka BP and follow a downward trend after the mid-Holocene. The same is true of the summer lamination thickness record, albeit this continued to increase throughout the mid-Holocene and decreases from 4 ka BP.

At Nautajärvi there is a good coherence between the reconstructed temperature data and pollen-based GDD (Ojala et al., 2008) and a regional July temperature comparison from Lake Kuutsjärvi (Salonen et al., 2024). The trends in the GDD reconstruction of Ojala et al. (2008) closely approximate those of the chironomid data. Further the pollen-based July temperature reconstruction from Lake Kuutsjärvi not only follows a very similar trend (peak warmth at ca. 6 ka BP) but also produces regional coherence in terms of absolute reconstructed temperatures (Salonen et al., 2024). As Diss Mere, the trends identified at Nautajärvi are closely associated with the Erb et al. (2022) regional data-assimilation approach.

The data from Meerfelder Maar are more difficult to substantiate as there are limited reconstructions from the Holocene from that record and few from Germany generally. Whilst pollen-based reconstructions of climate exist (e.g. Litt et al., 2009) the data show incredible stability for the Holocene period. We therefore rely on comparing to the transient simulations and data-assimilation approaches. Again the broad patterns/trends in the latter roughly correspond to the data we produce at Meerfelder Maar albeit with some offsets.

We therefore show that the data produced in this study is comparable to available data from each of the sites in this study. Where that isn't available, the data show a good match to regional reconstructions.

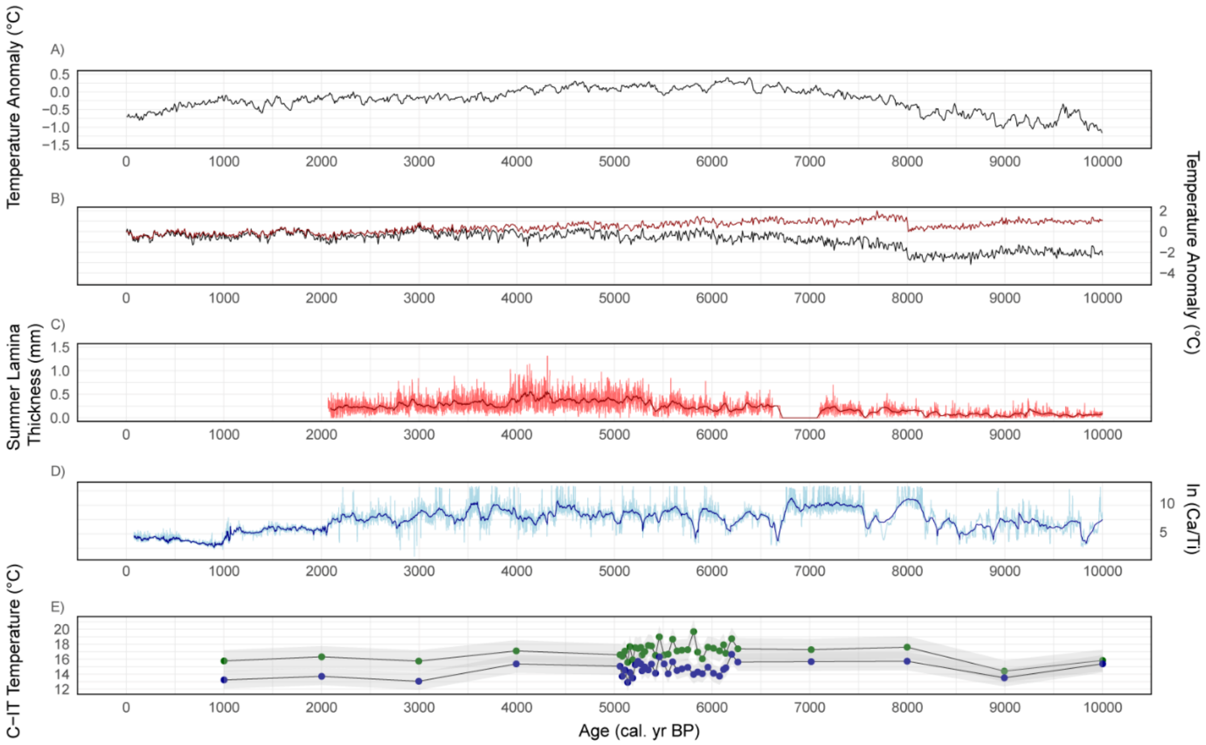

Figure S18. Comparisons of Diss Mere temperature reconstructions with additional available physical data from the lake, alongside regional climate observations. Shown are A) Holocene data-assimilation reconstruction anomalies between 50° and 60° (Erb et al., 2022); B) transient model simulation anomalies (TRaCE-21k) of mean annual temperature (black) and summer (JJA; red) for the 50° to 60° latitude band (Liu et al., 2009); C) annual calcite (summer) lamination thickness (light red) with a 50-year moving average applied (dark red) Martin-Puertas et al. (2021). Note the graph has been truncated to 1.5mm meaning one outlier at 4.338 ka BP, measuring 3.486mm is not shown; D) the ratio of calcium and titanium as measured across individual varve layers (light blue) with a 50-year moving average (dark blue) applied (Boyall et al., 2024); E) the two temperature reconstructions performed in this study. The Norwegian-Swiss reconstruction (green) and the Norwegian reconstruction (blue). Shown in panel E) are the individual sample specific errors associated with each reconstruction.

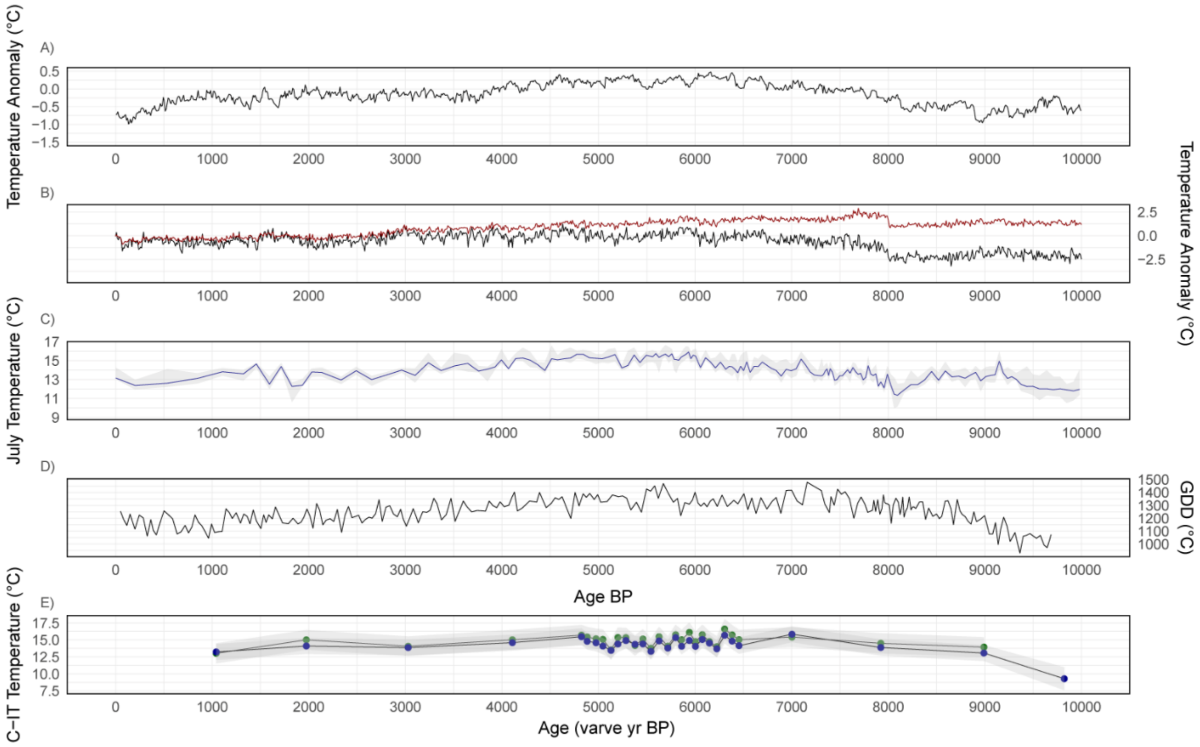

Figure S19. Comparisons of Nautajärvi temperature reconstructions with additional available physical data from the lake, alongside regional climate observations. Shown are A) Holocene data-assimilation reconstruction anomalies between 60° and 70° (Erb et al., 2022); B) transient model simulation anomalies (TRaCE-21k) of mean annual temperature (black) and summer (JJA; red) for the 60° to 70° latitude band (Liu et al., 2009); C) a pollen-based reconstruction of July temperatures using a six-method ensemble from Lake Kuutsjärvi, Finland, also shown are errors associated with 95% confidence intervals (Salonen et al., 2024); D) Pollen-derived growing degree days (GDD >5 °C) from Nautajärvi (Ojala et al., 2008); E) the two temperature reconstructions performed in this study. The Norwegian-Swiss reconstruction (green) and the Norwegian reconstruction (blue). Shown in panel E) are the individual sample specific errors associated with each timeseries.

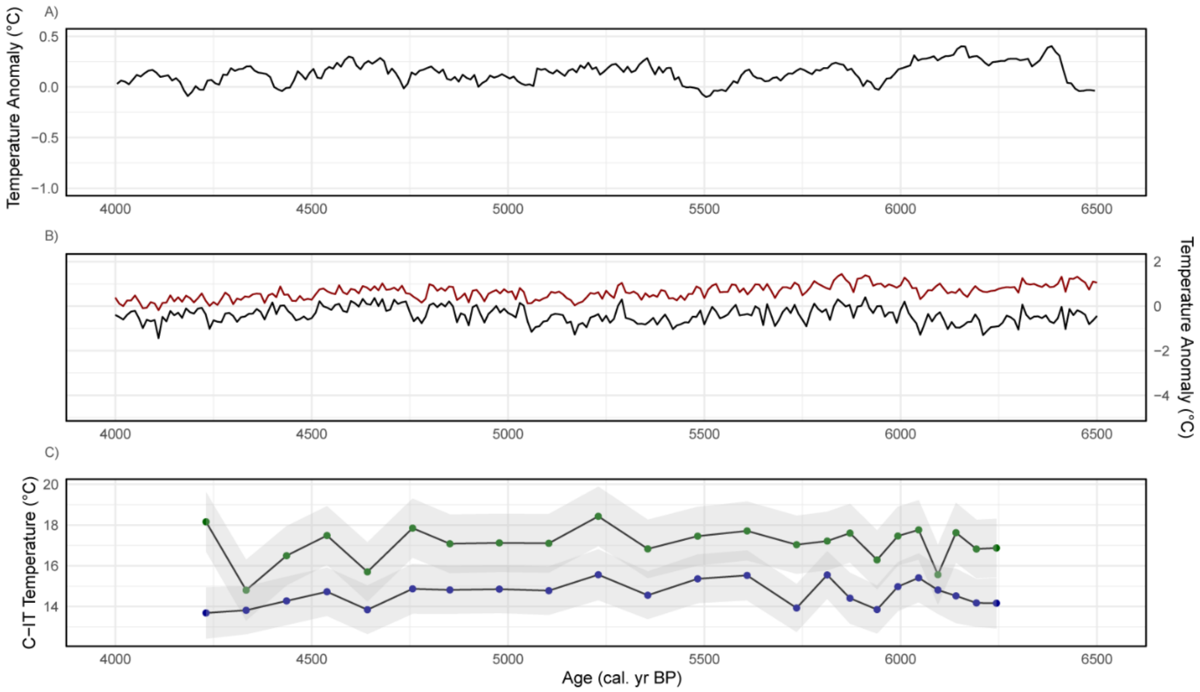

Figure S20. Comparisons of Meerfelder Maar temperature reconstructions with regional climate observations. Shown are A) Holocene data-assimilation reconstruction anomalies between 50° and 60° (Erb et al., 2022); B) transient model simulation anomalies (TRaCE-21k) of mean annual temperature (black) and summer (JJA; red) for the 50° to 60° latitude band (Liu et al., 2009); E) the two temperature reconstructions performed in this study. The Norwegian-Swiss reconstruction (green) and the Norwegian reconstruction (blue). Shown in panel E) are the individual sample specific errors associated with each timeseries.

References

Boyall, L., Martin-Puertas, C., Tjallingii, R., Milner, A.M., and Blockley, S.P. (2024). ‘Holocene climate evolution and human activity as recorded by the sediment record of lake Diss Mere, England.’ *Journal of Quaternary Science*, 39(6), pp. 972-986.

Engels, S., Medeiros, A.S., Axford, Y., Brooks, S.J., Heiri, O., Luoto, T.P., Nazarova, L., Porinchu, D.F., Quinlan, R., and Self, A.E. (2019). Temperature change as a driver of spatial patterns and long-term trends in chironomid (Insecta: Diptera) diversity. *Global Change Biology*, 26(3), pp.1155-1169.

Erb, M.P., McKay, N.P., Steiger, N., Dee, S., Hancock, C., Ivanovic, R.F., Gregoire, L.J., and Valdes, P. (2022). ‘Reconstructing Holocene temperatures in time and space using paleoclimate data assimilation.’ *Climate of the Past*, 18(12), pp. 2599-2629.

Litt, T., Schölzel, C., Kühl, N., and Brauer, A. (2009). ‘Vegetation and climate history in the Westeifel Volcanic Field (Germany) during the past 11 000 years based on annually laminated lacustrine maar sediments.’ *Boreas*, 38(4), pp. 679-690.

Liu, Z., Otto-Bliesner, B.L., He, F., Brady, E.C., Tomas, R., Clark, P.U., Carlson, A.E., Lynch-Stieglitz, J., Curry, W., Brook, E., Erickson, D., Jacob, R., Kutzbach, J., and Cheng, J. (2009). ‘Transient simulation of last deglaciation with a new mechanism for Bølling-Allerød warming.’ science, 325(5938), pp. 310-314.

Martin-Puertas, C., Walsh, A.A., Blockley, S.P., Harding, P., Biddulph, G.E., Palmer, A., Ramisch, A., and Brauer, A. (2021). ‘The first Holocene varve chronology for the UK: Based on the integration of varve counting, radiocarbon dating and tephrostratigraphy from Diss Mere (UK).’ *Quaternary Geochronology*, 61, pp. 101134.

Ojala, A.E., Alenius, T., Seppä, H., and Giesecke, T. (2008). ‘Integrated varve and pollen-based temperature reconstruction from Finland: evidence for Holocene seasonal temperature patterns at high latitudes.’ *The Holocene*, 18(4), pp. 529-538.

Oksanen, J., Simpson, G., Blanchet, F., Kindt, R., Legendre, P., Minchin, P., O'Hara, R., Solymos, P., Stevens, M., Szoecs, E., Wagner, H., Barbour, M., Bedward, M., Bolker, B., Borcard, D., Carvalho, G., Chirico, M., De Caceres, M., Durand, S., Evangelista, H., FitzJohn, R., Friendly, M., Furneaux, B., Hannigan, G., Hill, M., Lahti, L., McGlinn, D., Ouellette, M., Ribeiro Cunha, E., Smith, T., Stier, A., Ter Braak, C., and Weedon, J. (2024). ‘Vegan: Community Ecology Package.’ R package version 2.6-6.1 [Sotfware]. https://CRAN.R-project.org/package=vegan.

Salonen, J.S., Kuosmanen, N., Alsos, I.G., Heintzman, P.D., Rijal, D.P., Schenk, F., Bogren, F., Luoto, M., Philip, A., Piilo, S., and Trasune, L. (2024). ‘Uncovering Holocene climate fluctuations and ancient conifer populations: Insights from a high-resolution multi-proxy record from Northern Finland.’ *Global and Planetary Change*, *237*, pp.104462.

Simpson, G.L. (2007). ‘Analogue methods in palaeoecology: using the analogue package.’ *Journal of Statistical Software*, 22, pp. 1-29.

Simpson, G.L., Oksanen, J., and Maechler, M. (2024). ‘analogue: Analogue and Weighted Averaging Methods for Palaeoecology.’ R package version 0.17-7 [Software]. 10.32614/CRAN.package.analogue.
